# Engineering small peptide secretion in *Komagataella phaffii*

**DOI:** 10.3389/fbioe.2026.1929744

**Published:** 2026-09-16

**Authors:** Johanna Pirchner, Simon Arhar, Corinna Odar, Lisa Frühauf, Azra Kapetanović, Uma Isaković, Susanne Lux, Sven Heimhilcher, Andreas Winkler, Anton Glieder, Anita Emmerstorfer-Augustin

**Affiliations:** 1 Institute of Molecular Biotechnology, Graz University of Technology, NAWI Graz, Graz, Austria; 2 Austrian Centre of Industrial Biotechnology, acib GmbH, Graz, Austria; 3 Institute of Chemical Engineering and Environmental Technology, Graz University of Technology, Graz, Austria; 4 bisy GmbH, Hofstätten ander Raab, Austria; 5 Institute of Biochemistry, Graz University of Technology, NAWI Graz, Graz, Austria; 6 BioTechMed-Graz, Graz, Austria

**Keywords:** ER translocon, knockout screening, Komagataella phaffii, secretion, signal sequence, small peptides

## Abstract

**Background:**

Small peptides have emerged as an important class of biologics with broad therapeutic applications. Limitations of conventional solid-phase synthesis have intensified the interest in microbial production systems. *Komagataella phaffii*, a widely used host for secretory protein production, represents a promising alternative platform; however, the efficient secretion of very small peptides (<3.5 kDa) remains a major biotechnological challenge due to constraints in detection, proteolytic processing, and overall secretion efficiency.

**Methods:**

To systematically investigate bottlenecks in small peptide secretion, we established the 3×FLAG peptide (23 amino acids) as a tractable, immunologically detectable model system. We evaluated the impact of peptide chain length and signal sequence selection on secretory performance. Furthermore, we assessed the effect of overexpressing the translocon components and conducted a random mutagenesis screen to identify novel genetic determinants influencing secretion. Finally, we tested the transferability of our engineering strategies on the disulfide-rich peptide dQ-brazzein and the therapeutic fusion peptides α-melanocyte-stimulating hormone and salmon calcitonin.

**Results:**

Secretion efficiency exhibited a strong dependency on peptide chain length and signal sequence selection, likely reflecting limitations during endoplasmic reticulum translocation. Overexpression of Sec61 partially alleviated this bottleneck, enhancing secretion approximately 2-fold. Through the random mutagenesis screen, we identified *AQR1*, *FMS1-2*, *SKG3*, and *CDC48* as novel targets, which increased secretion up to 9-fold. These engineering strategies were tested on dQ-brazzein, yielding up to ∼100% improvement, though the magnitude of transfer was cargo-dependent. Furthermore, the pronounced secretion limitations observed for α-melanocyte-stimulating hormone and salmon calcitonin based fusion peptides were partially mitigated utilizing these strategies, resulting in a ∼2-fold improvement.

**Conclusion:**

This study provides a systematic evaluation of engineering strategies targeting the unique constraints of very small (<3.5 kDa) peptide secretion in *K. phaffii*. The identified genetic targets and mechanistic insights serve as a starting point for cargo-specific optimization efforts and highlight the unique challenges that short peptides pose to the native secretory machinery.

## 1 Introduction

Small peptides (≤100 amino acids) are gaining increasing attention as potent therapeutic agents and valuable biotechnological tools, owing to their high target specificity, low immunogenicity, and ability to engage challenging biological targets (Muttenthaler et al., 2021). The growing demand for peptide-based products is driving efforts to establish more efficient, scalable, and sustainable production methods. In this context, microbial biosynthesis has emerged as a promising production platform, with production by *Komagataella phaffii* attracting particular interest. This methylotrophic yeast is widely used in industrial biotechnology due to its strong protein synthesis capabilities, ease of genetic manipulation, and capacity for post-translational modifications such as glycosylation (Ahmad et al., 2014; Bernauer et al., 2021). Yet, till now production of small peptides, especially below 3.5 kDa in size is challenging and the reported titers do not compare to the ones achievable with larger proteins (Werten et al., 1999; Chang-Chih et al., 2004; Gratzer et al., 2026).

A key advantage of *K. phaffii* is the ability to secrete peptides directly into the culture medium, which greatly simplifies downstream processing. Two main strategies have been established for the secretory production of small peptides. The first involves expression of tandem peptide repeats separated by protease recognition sequences, which are subsequently cleaved to release the mature peptide. Examples include the antifungal peptide CGA-N12 (1.3 kDa, 32.65 ng/L), expressed as four tandem copies flanked by Kex2 and Ste13 sites (Li et al., 2020); the ginseng-derived peptide GSP (1.4 kDa), produced in a four-copy tandem format with Kex2 cleavage sites (Yan et al., 2003); and the beefy-meaty peptide (0.85 kDa), expressed as up to 16 linked repeats separated by 4VE sequences (Wang et al., 2011). However, this strategy is complicated by incomplete cleavage or the retention of residual amino acids at peptide termini, which can compromise biological activity and regulatory acceptance.

The second, more streamlined strategy is direct secretion of single-copy peptides via N-terminal signal sequence fusion. This approach has been demonstrated for enterocin L50 (5 kDa) (Basanta et al., 2010), the plectasin derivative NZ2114 (4.4 kDa, 2,390 mg/L) (Zhang et al., 2014), apidacein (2.2 kDa) (Chen et al., 2017), thanatin (2.4 kDa, reported estimate of 20 mg/L) (Pipiya et al., 2023) and hepcidin (2.2 kDa) (Tian et al., 2016), all driven by the strong methanol-inducible *AOX1* promoter. Nevertheless, production levels were extremely low in many cases, requiring detection and purification via affinity tags. The MFα signal sequence remains the most commonly used and broadly effective option for protein and peptide secretion in *K. phaffii* (Merkaš et al., 2025), yet it carries notable limitations. In particular, the EAEA (Glu-Ala-Glu-Ala) spacer, intended for removal by Ste13 following Kex2 cleavage, is often only partially processed, leaving unwanted N-terminal residues on the mature peptide and potentially reducing bioactivity. To circumvent this, many studies omit the EAEA spacer and rely solely on Kex2 cleavage (Neiers et al., 2021; Raemaekers et al., 1999), though this can lead to reduced secretion efficiency and heterogeneous N-termini depending on the peptide’s P1’ residue. Alternative signal sequences such as Ost1 have demonstrated more precise N-terminal processing in specific contexts, for example for lytic polysaccharide monooxygenases (LPMOs), where correct processing of the N-terminal histidine is essential for activity (Rieder et al., 2021). Additional signal sequences reported to undergo efficient and correct processing include *K. phaffii* Exp1, *K. phaffii* S30, and *K. phaffii* Scw10 (Heiss et al., 2015; Shen et al., 2022).

Signal peptides direct proteins into the secretory pathway via the endoplasmic reticulum (ER) and Golgi apparatus, using either co-translational or post-translational translocation. The MFα signal sequence typically engages the post-translational route, while Ost1 drives co-translational translocation, a mechanism dominant in mammalian cells and generally efficient for larger proteins. In mammalian systems, however, short peptides are often poorly translocated via the co-translational pathway due to limited recognition by the signal recognition particle (SRP), increasing the risk of cytosolic degradation or misprocessing. Recent work indicates that small proteins and peptides (≤100 amino acids) can be efficiently secreted via a Sec62-dependent post-translational pathway (Lakkaraju et al., 2012). While Sec61 forms the core protein-conducting channel of the ER translocon, Sec62 acts as an auxiliary component that facilitates post-translational import of short polypeptides, suggesting that the two subunits play distinct but complementary roles in small peptide translocation. This raises a fundamental question for yeast systems: is there a minimum size threshold for efficient secretion in *K. phaffii*? Prior work has addressed secretion of individual small peptides in *K. phaffii* (e.g. apidaecin, hepcidin, enterocin L50), yet a systematic evaluation of the specific constraints governing sub-3.5 kDa peptide secretion and a genetic parts list for overcoming them has been lacking. Synthesis of short peptides is completed rapidly, potentially before co-translational translocation machinery can engage.

In this study, we established stable secretion of the 3×FLAG peptide, a small, immunologically detectable model peptide, in *K. phaffii*. The production strategy was based on the strong methanol-inducible *AOX1* promoter and utilized methanol produced from CO_2_, emphasizing on the recently renewed interest in methanol utilization due to advances in carbon capture and utilization technologies. By selecting signal sequences with demonstrated processing fidelity, we determined that peptides as short as 16 amino acids can be secreted using the *S. cerevisiae* Ost1 signal sequence. Although initial yields were low, secretion was markedly improved by overexpression of *SEC61*, a core component of the ER translocon. Complementary to this, we applied restriction enzyme-mediated integration (REMI) mutagenesis (Schroder et al., 2007) to identify gene deletions that further enhance peptide secretion. The relevance of these findings extends beyond the 3×FLAG model: improved secretion was also partially demonstrated for the peptide sweetener dQ-brazzein (6.5 kDa) (Poirier et al., 2012) and two fusion peptides based on the therapeutic hormone calcitonin and α-melanocyte-stimulating hormone (α-MSH), underscoring the potential applicability of the strategies described here, but also highlighting differences in production limitations between target peptides.

## 2 Materials and methods

### 2.1 Cloning and strain construction

Plasmids used in this study (Supplementary Table S1) were constructed by Gibson Assembly (Gibson et al., 2009) using the primers listed in Supplementary Table S2 and verified by restriction analysis and Sanger sequencing. *E. coli* Top10 cells used during plasmid construction were cultivated in LB medium at 37 °C with the appropriate antibiotic (Ampicillin, 100 μg/mL; Zeocin, 100 μg/mL; or Hygromycin B, 200 μg/mL). Phusion polymerase and Fast-Digest restriction enzymes (Thermo Fisher Scientific) were used for all cloning steps according to the manufacturer’s instructions.

*K. phaffii* CBS7435 *his4*Δ (Näätsaari et al., 2012) was used to construct the 3×FLAG production strain. The prototrophic *K. phaffii* BSYBG10 strain, derived from *K. phaffii* NRRL Y-11430 by curing of killer plasmids and supplied by bisy GmbH (Hofstätten an der Raab, Austria) (Sturmberger et al., 2016), was used to generate production strains for dQ-brazzein, CalB, salmon calcitonin, and α-MSH. *K. phaffii* transformation was performed as previously described (J. Lin-Cereghino et al., 2005). Positive transformants were selected on YPD agar plates (10 g/L yeast extract, 20 g/L peptone, 20 g/L dextrose, 20 g/L agar) supplemented with the appropriate antibiotic (Zeocin, 100 μg/mL; or Hygromycin B, 200 μg/mL). All strains generated in this study are listed in Supplementary Table S3.

Expression plasmids for small peptide production were constructed by digesting the pPpT4-αS plasmid with *Eco*RI and *Not*I. The sequences encoding 3×FLAG, dQ-brazzein, α-MSH, and salmon calcitonin, together with the respective signal sequences and assembly overlaps, were ordered as double-stranded DNA fragments (IDT) and assembled by Gibson cloning. For *K. phaffii* transformation, 100 ng of *Smi*I-linearized plasmid was used per transformation.

For testing 3xFLAG secretion with different signal sequences and secretion of truncated variants, expression cassettes were integrated into the *AOX1* locus, as verified by colony PCR.

Vectors for overexpression of ER translocon components were generated by overlap extension PCR to combine the hygromycin resistance cassette with the respective expression cassettes. *SEC61*, *SEC62*, *SEC63*, and *KAR2* were amplified from *K. phaffii* CBS7435 genomic DNA (Supplementary Table S4).

The pREMIh plasmid was constructed by replacing the resistance cassette of pREMI-ZA (Ito et al., 2022) with a hygromycin resistance cassette. The pREMI-ZA plasmid was digested with *Nde*I and *Nco*I, and the hygromycin resistance cassette was amplified by PCR and inserted. Targeted gene knockouts were performed using a CRISPR/Cas9-based genome editing system (Lehmayer et al., 2022), adapted from Weninger et al. (2018). Gene-specific protospacer sequences were introduced into the pPpHyg-Cas9 plasmid by PCR using the primers listed in Supplementary Table S2. For each knockout, 100 ng of the CRISPR/Cas9 plasmid and 200 ng of a repair oligonucleotide were co-transformed to introduce the desired mutation. Engineered *K. phaffii* strains were verified using the Phire Plant Direct PCR Kit (Thermo Fisher Scientific) followed by Sanger sequencing.

For CalB production, a previously published pPpT4-αS-based plasmid (Vogl et al., 2016) was linearized with *Smi*I and 100 ng used for transformation.

### 2.2 Media and cultivation conditions

#### 2.2.1 Deep-well plate (DWP) cultivations

For screening, *K. phaffii* clones were cultivated in sterile 96-deep-well plates. Each well was filled with 300 µL BMD medium (13.4 g/L yeast nitrogen base without amino acids (Sigma Aldrich), 200 mM potassium phosphate buffer pH 6, 0.4 mg/L biotin, 20 g/L dextrose, supplemented with 40 mg/L histidine where required) and inoculated with a single colony using a sterile toothpick. Cultures were incubated at 28 °C and 320 rpm for approximately 60 h. Induction was initiated by adding 300 µL BMM1 medium (13.4 g/L yeast nitrogen base without amino acids, 200 mM potassium phosphate buffer pH 6, 0.4 mg/L biotin, 0.5% (v/v) methanol). After approximately 12 h, an additional 60 µL BMM10 (5% (v/v) methanol) was added. After 24 h of induction, cells and culture supernatant were harvested by centrifugation at 3,200 × *g* for 10 min at 4 °C.

#### 2.2.2 Shake flask cultivations

Pre-cultures were grown overnight in 3 mL YPD at 28 °C and 130 rpm. Main cultures were inoculated into 100 mL baffled shake flasks containing 10 mL BMD at an initial OD_600_ of 0.1 and incubated at 28 °C and 130 rpm for approximately 60 h. For induction, cultures were transferred to sterile tubes, centrifuged at room temperature for 5 min at 1,100 × g, and the pellets resuspended in 10 mL BMM1 before being returned to the shake flasks. Crucially, 100% of the methanol used for both DWP and shake flask cultivations throughout this entire study was exclusively derived from captured CO_2_. This sustainable synthesis route utilized Cu/MgO catalysts for CO_2_ hydrogenation (Kleiber et al., 2021). Induction was maintained by supplementing this CO_2_-derived methanol to 0.5% (v/v) every 12 h. Cultures were harvested after 24 h (3×FLAG, α-MSH, and salmon calcitonin) or 48 h (dQ-brazzein and CalB) post-induction by centrifugation at 4 °C and 3,200 × g for 10 min. Supernatants and cell pellets were either processed immediately or stored at −20 °C.

### 2.3 Screening methods

#### 2.3.1 pREMIh screening

For random knockout screening, pREMIh was linearized with either *Bam*HI or *Kpn*I. The *K. phaffii* CBS7435 *his4Δ P*_AOX1_-*OST1-3×FLAG* strain was transformed with 300 ng of the linearized construct and selected on YPD agar supplemented with hygromycin. Random knockout clones were cultivated in deep-well plates and harvested after 24 h of induction. Culture supernatants were analyzed immediately by dot blot or stored at −20 °C.

The REMI isolation protocol was adapted from Ito et al. (2022). Briefly, clones showing improved 3×FLAG secretion were grown overnight in 3 mL YPD containing hygromycin, harvested by centrifugation at 3,200 × *g* for 5 min, and washed once with ddH_2_O. Cell pellets were resuspended in 200 µL yeast lysis buffer (50 mM Tris/HCl pH 7.2, 50 mM EDTA, 3% SDS, 1% β-mercaptoethanol), followed by addition of 200 µL phenol–chloroform–isoamyl alcohol (25:24:1) and 100 µL glass beads (0.3–0.5 mm). Samples were vortexed at maximum speed for 5 min. After adding 200 μL TE buffer (10 mM Tris/HCl pH 8.0, 1 mM EDTA), samples were centrifuged at 16,000 × *g* for 5 min at 4 °C. The aqueous phase was transferred to a new tube, and genomic DNA was precipitated with 1 mL ice-cold 100% ethanol, pelleted by centrifugation (16,000 × *g*, 5 min, 4 °C), washed with 1 mL 100% ethanol, air-dried at 37 °C for 30–60 min, and resuspended in 400 μL TE. RNA was removed by overnight incubation at 37 °C with 5 µL RNase A (10 mg/mL). DNA was then precipitated with 1 mL ice-cold 100% ethanol and 10 µL 4 M ammonium acetate, pelleted, washed twice with 70% ethanol, air-dried, and dissolved in 50 µL molecular-grade ddH_2_O. DNA concentration and purity were assessed spectrophotometrically.

Approximately 5 µg of genomic DNA was digested with a combination of FastDigest restriction enzymes (*Apa*I, *Nhe*I, *Eco*RV, and *Xho*I, 1 µL each) in a total volume of 50 μL at 37 °C for 4 h, followed by heat inactivation at 75 °C for 10 min. Fragment sizes were confirmed by agarose gel electrophoresis. For blunt-end generation, 10 µL of digested DNA was treated with 2 mM dNTPs and 0.2 µL Klenow fragment (Thermo Fisher Scientific) in the presence of T4 DNA ligase buffer in a 20 µL reaction at 37 °C for 10 min, followed by inactivation at 75 °C for 10 min. For ligation, 1 µL T4 DNA ligase and 2 µL PEG 4000 were added and the reaction incubated at 22 °C for 5 h. After desalting, 5 µL of the ligation product was transformed into *E. coli* Top10 cells and plated on LB agar with ampicillin. Transformants were replica-plated onto LB–hygromycin and LB–ampicillin plates; only colonies growing under both selection conditions were used for plasmid isolation. Plasmids were sequenced and the *K. phaffii* genomic integration loci identified by nucleotide BLAST (Camacho et al., 2009).

#### 2.3.2 Dot blot screenings

Dot blots were performed on nitrocellulose membranes (0.45 µm pore size). Membranes were mounted in the dot blot apparatus and prewashed with 100 µL TBS (3 g/L Tris base, 8.8 g/L NaCl, pH 7.0) per well under vacuum. For sample application, 50 µL TBS and 250 μL *K phaffii* culture supernatant were loaded per well and incubated for 10 min at room temperature before vacuum was applied. Membranes were air dried for 5 min and then washed with 200 µL TBS and air-dried for 5 min again. Blocking was performed in TBST-BSA (TBS supplemented with 0.003% Tween-20% and 5% BSA) for 3 h at room temperature with gentle agitation. ANTI-FLAG® M2 peroxidase (HRP) antibody (Sigma-Aldrich, A8592; 1:2500 in TBST with 2.5% BSA) was applied overnight at 4 °C with gentle shaking.

### 2.4 Protein analysis

#### 2.4.1 Sequence alignments and structure predictions

Multiple sequence alignments were generated using Clustal Omega (Sievers et al., 2011) and visualized with pyBoxshade (https://github.com/mdbaron42/pyBoxshade). Peptide structures were predicted using the AlphaFold3 Server (Abramson et al., 2024).

#### 2.4.2 SDS-PAGE

To assess the intracellular fraction of the 3×FLAG peptide, cell lysis was performed as previously described (Horvath and Riezman, 1994). Briefly, cells from 4 OD_600_ units were harvested by centrifugation, resuspended in 300 µL lysis solution (1.85 M NaOH, 7.5% β-mercaptoethanol), and incubated on ice for 10 min. Proteins were precipitated by adding 300 µL 50% (w/v) trichloroacetic acid (TCA) and incubating at 4 °C for 1 h. After centrifugation (16,000 × *g*, 5 min, 4 °C), the pellet was washed with 1 mL ice-cold water and resuspended in 50 µL 1× NuPAGE LDS sample buffer (Thermo Fisher Scientific).

Culture supernatants were precipitated by methanol/chloroform extraction. Unless otherwise stated, 250 µL supernatant was mixed with 380 µL methanol, 160 µL chloroform, and 490 µL ddH_2_O on ice, vortexed for 30 s, and centrifuged (16,000 × *g*, 5 min, 4 °C). The upper aqueous phase was discarded, 300 µL methanol was added, and the sample was vortexed and centrifuged again. The resulting pellet was air-dried for ∼30 min and resuspended in 30 µL 1× NuPAGE LDS sample buffer containing 2% β-mercaptoethanol. Samples were heated at 70 °C for 10 min and 15 µL loaded per lane.

SDS-PAGE was performed using NuPAGE 4%–12% Bis-Tris Mini Protein Gels (1.0 mm; Thermo Fisher Scientific). Samples of 3×FLAG, dQ-brazzein, α-MSH, and salmon calcitonin were run in MES buffer with the Spectra™ Multicolor Low Range Protein Ladder (Thermo Fisher Scientific). CalB samples were run in MOPS buffer with the PageRuler Prestained Protein Ladder (Thermo Fisher Scientific).

#### 2.4.3 Immunoblot analysis

Proteins were transferred to nitrocellulose membranes (0.22 µm pore size) using a wet blotting system (NuPAGE, Thermo Fisher Scientific). Membranes were blocked in TBST with 5% BSA for 3 h at room temperature with gentle agitation, then incubated with ANTI-FLAG M2 Peroxidase (HRP) antibody (Sigma-Aldrich; 1:1500 in TBST with 2.5% BSA) overnight at 4 °C. After three washes with TBST (5 min each), signal was detected using the Clarity Max Western ECL Substrate (Bio-Rad) and a G:Box imaging system (Syngene).

#### 2.4.4 Relative Quantification of SDS-PAGE and immunoblots

Signals corresponding to the target protein band were quantified by densitometry using the Fiji software (Schindelin et al., 2012). For relative quantification, extracted peak areas corresponding to the target bands were compared to the parental production strain, which was present on each blot or gel as control.

#### 2.4.5 Mass spectrometry

Culture supernatants were either directly subjected to intact mass measurements or pre-processed by filtration. Where necessary for buffer exchange or peptide concentration, Amicon Ultra-4 Ultracel-3 kDa centrifugal filters (Millipore) were used according to the manufacturer’s instructions. For TEV protease cleavage, samples were buffer-exchanged into 50 mM Tris/HCl (pH 7.5) containing 1 mM DTT and 0.5 mM EDTA. Cleavage was initiated by adding 10 units TEV protease (GenScript, Z03030) in a 200 µL reaction and incubated overnight at 4 °C. Secreted peptides were compared to commercial standards: 3×FLAG (Sigma Aldrich, F4799), dQ-brazzein (custom synthesis, GenScript), α-MSH (GenScript, RP10644-5), and salmon calcitonin (Sigma Aldrich, T3360).

Intact mass measurements were performed on an Impact II UHR-TOF mass spectrometer (Bruker). Aliquots of 3 μL at 10 µM were desalted on a Shim-pack Scepter C4-300 (G) column (3 μm; Shimadzu) by washing with 1% acetonitrile and 0.1% formic acid, followed by elution with an acetonitrile gradient (1%–95%) into an Impact II ESI-Q-TOF (Bruker). Spectra were integrated and deconvoluted using the maximum entropy or small peptide function of DataAnalysis (Bruker). Quantitative evaluations of the spectra were based on the extracted ion chromatogram of standards and samples.

#### 2.4.6 CalB activity assay

CalB activity in culture supernatant was measured as previously described (Krainer et al., 2012). Briefly, 20 µL supernatant was mixed with 180 µL assay solution (4 mM *p*-nitrophenyl butyrate, 300 mM Tris/HCl pH 7.4, 1% acetone) and the increase in absorbance at 405 nm was monitored at room temperature for 15 min using a BioTek Synergy MX microplate reader. One unit of lipase activity was defined as the amount of enzyme converting 1 µmol substrate per minute. All assays were performed in technical triplicates from two independent biological replicates.

## 3 Results

### 3.1 Establishing 3×FLAG as a model for small peptide secretion

Small peptides below 3.5 kDa present an analytical challenge: they are typically secreted in low amounts and require expensive, complex detection methods such as mass spectrometry. To bypass this limitation, we used the 3×FLAG peptide as a model for small peptide secretion in *K. phaffii*. The 3×FLAG peptide contains an epitope tag that enables immunological detection without additional affinity tags and can be stepwise truncated while retaining detectability. At 23 amino acids, the 3×FLAG peptide falls within the size range of many therapeutically relevant peptide hormones (Wang et al., 2022).

To identify a suitable signal sequence, we compared the *S. cerevisiae* Ost1_pre_ signal (Fitzgerald and Glick, 2014; Rieder et al., 2021), the MFα_prepro_ signal (Invitrogen Variant (Merkaš et al., 2026)) lacking the EAEA spacer, and the *K. phaffii* S30_pre_, Exp1_pre_, and Scw10_prepro_ signals (Heiss et al., 2015; Shen et al., 2022), all previously reported to undergo efficient and correct processing (Figure 1A). Immunoblot analysis revealed that only the *S. cerevisiae* Ost1 signal sequence supported detectable secretion of the 3×FLAG peptide into the culture supernatant; all other signal sequences yielded no or negligible amounts of extracellular peptide (Figure 1B). The secreted band migrated at an apparent molecular weight of ∼10 kDa, despite the predicted mass of ∼2.9 kDa for the 3×FLAG peptide. This anomalous migration is consistent with the phenomenon of disproportionate SDS binding by small, highly acidic peptides, which results in an apparent molecular weight significantly exceeding the predicted mass. (Tiwari et al., 2019). Mass spectrometry confirmed the correct amino acid composition of the secreted product (Supplementary Figure S1A/B). AlphaFold3 structure prediction (Abramson et al., 2024) indicated that in contrast to many other small peptides, the 3×FLAG peptide adopts a largely unstructured conformation (Figure 1C).

**FIGURE 1:**
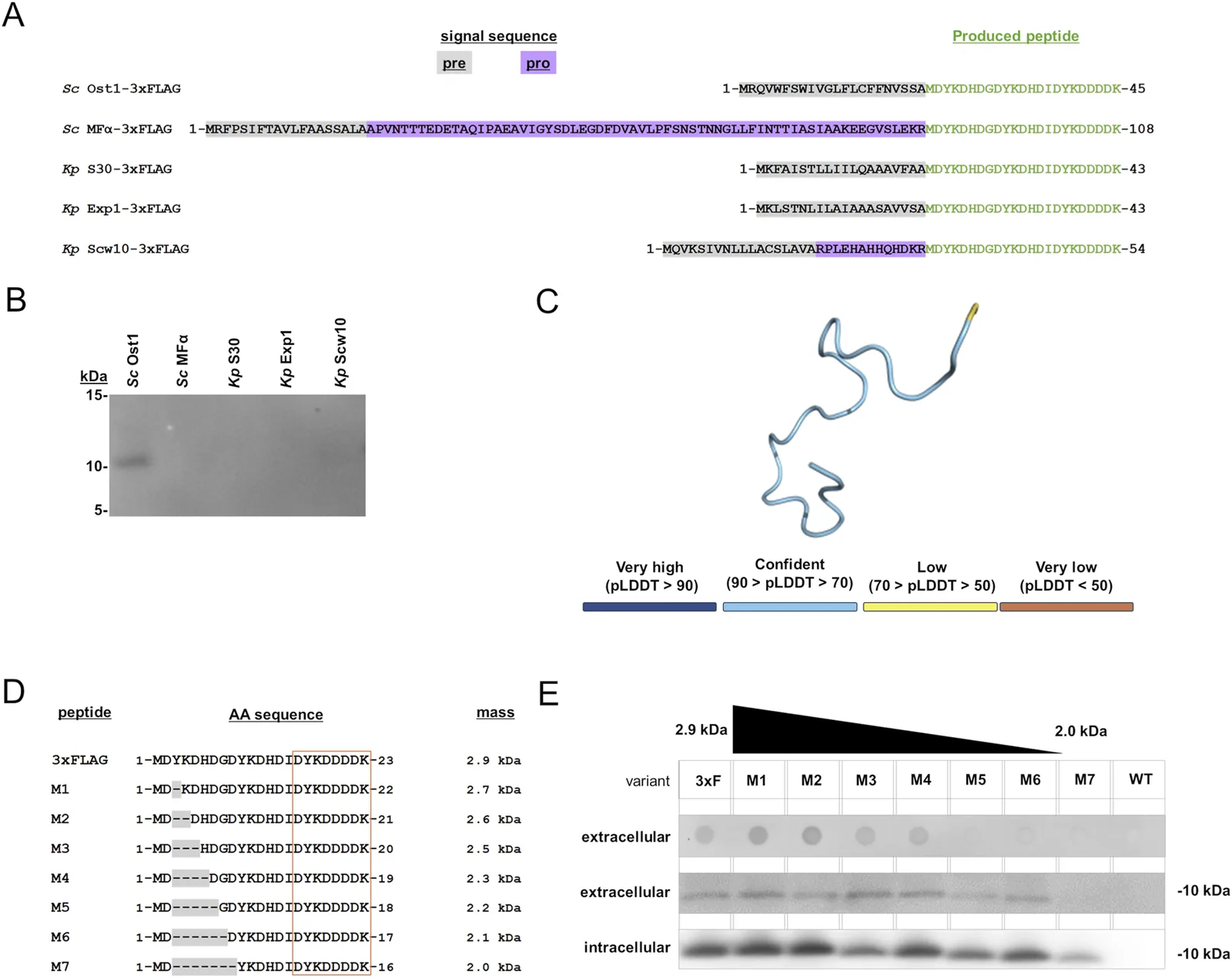
Secretion and size dependence of the 3×FLAG peptide in *K. phaffii*. **(A)** Signal peptide sequences tested in fusion with the 3×FLAG sequence: *S. cerevisiae* MFα (prepro) without C-terminal EAEA (Invitrogen variant L42S, D83E), *S. cerevisiae* Ost1 (pre), *K. phaffii* S30 (pre), *K. phaffii* Scw10 (prepro), and *K. phaffii* Exp1 (pre). **(B)** Anti-FLAG immunoblot analysis of culture supernatants from strains expressing 3×FLAG fused to the different signal sequences. Strains were constructed by target vector integration into the *AOX1* locus. Cells were cultivated in shake flasks for 60 h in BMD before induction for 24 h using methanol. For immunoblot analysis, samples corresponding to 125 µL of culture supernatant was subjected to methanol/chloroform precipitation. **(C)** AlphaFold3-predicted structure of 3×FLAG including the color coded predicted local distance difference (pLDDT). **(D)** Sequences of truncated 3×FLAG variants expressed with an N-terminal Ost1_pre_ signal sequence to determine size dependency of secretion. The box highlights the epitope sequence recognized by the Anti-FLAG antibody. **(E)** Size cut-off analysis of secretion efficiency for truncated 3×FLAG variants. Shown are the full-length 3×FLAG and the variants M1-M7 spanning a size of 2.9–2.0 kDa. All target construct were integrated at the *AOX1* locus. Expression of the different variants was analyzed in the culture supernatant via dot blot (line 1, extracellular) or via immunoblots using Anti-FLAG antibody. Immunoblots were performed on precipitated culture supernatant (line 2, extracellular) or on chemically lysed cells (line 3, intracellular, loaded sample corresponds to 1 OD_600_). Cells were cultivated and samples prepared as described for section **(B)**.

We next examined the effect of peptide size on secretion efficiency by stepwise truncation of the 3×FLAG construct by seven amino acids (Figure 1D), while retaining antibody detectability via the C-terminal epitope. Secretion efficiency decreased with each truncation step. Intracellular peptide levels also declined in parallel (Figure 1E), indicating that limitations arise not only during secretion.

Importantly, no difference in 3×FLAG secretion was observed between cultivations using CO_2_-derived and commercially available fossil-derived methanol (Supplementary Figure S1), demonstrating that sustainable methanol can serve as a direct drop-in replacement for conventional methanol in *K. phaffii* bioprocesses without compromising recombinant peptide production.

### 3.2 ER translocation as a target for engineering small peptide secretion

Given the observed intracellular accumulation of 3×FLAG (Figure 1E), we investigated whether overexpressing key translocon components could enhance extracellular peptide levels (Figure 2A). Linear vectors driving constitutive (*HHX1* promoter) overexpression of *SEC61*, *SEC62*, *SEC63*, or *KAR2*, individually or in combination for *SEC61* and *SEC62*, were integrated randomly into the genome to cover different expression levels due to copy number and locus effects. Of these, only overexpression of the central translocon pore *SEC61* produced an approximately 2-fold increase in 3×FLAG secretion across multiple independent clones (Figure 2B). Overexpression of the auxiliary factors *SEC62*, *SEC63*, or the ER chaperone *KAR2* did not consistently improve secretion.

**FIGURE 2:**
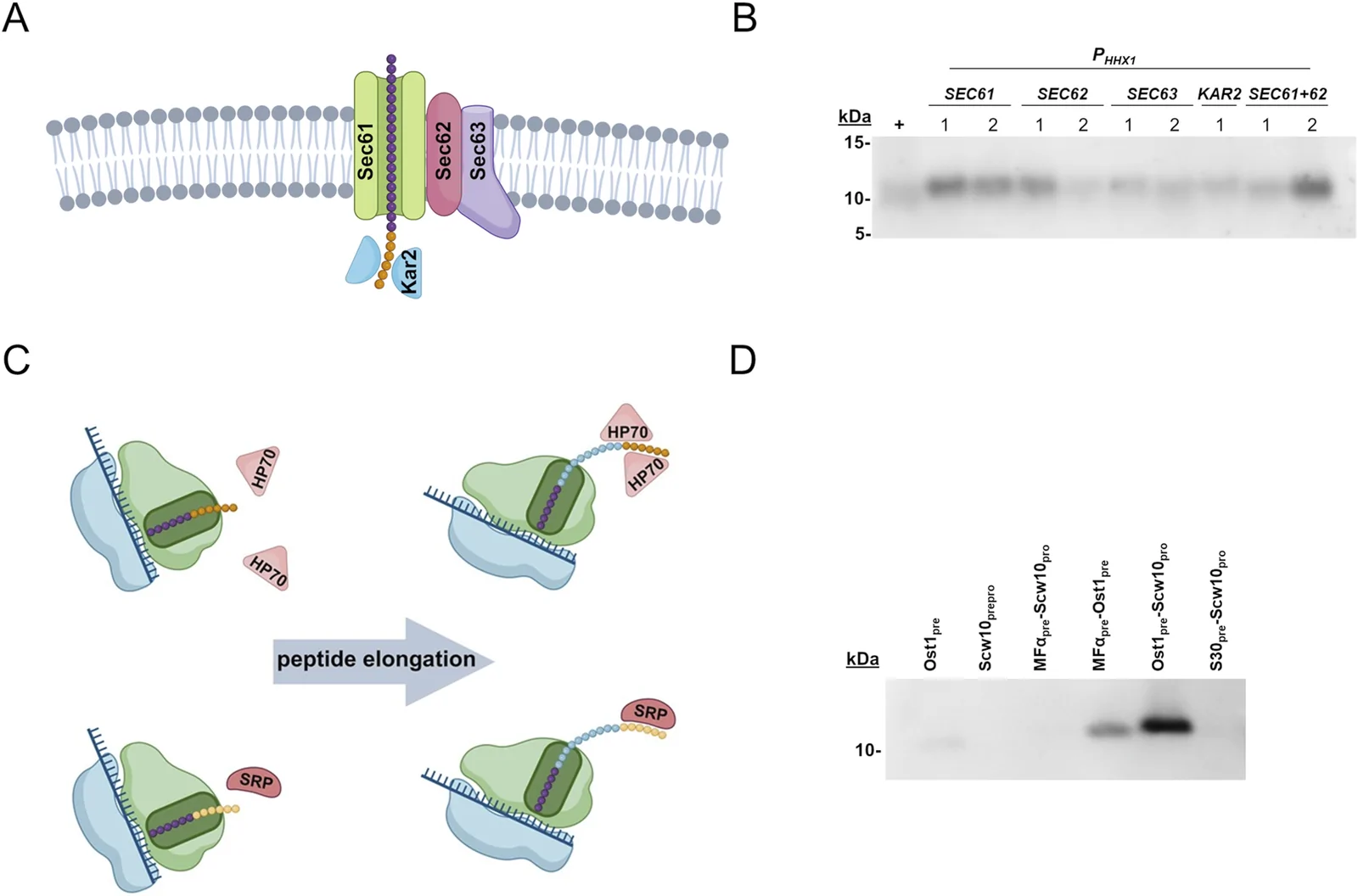
Translocon pore availability and signal sequence architecture influence 3×FLAG secretion. **(A)** Schematic of translocon components overexpressed under the constitutive *HHX1* promoter to increase 3×FLAG secretion. **(B)** Immunoblot analysis of culture supernatants from two independent clones per overexpression construct using Anti-FLAG antibody. The parental Ost1_pre_-3×FLAG secretion strain was added as control (+). *K. phaffii* strains were cultivated in shake flasks using BMD for biomass accumulation during a 60 h batch phase. 3×FLAG expression was induced using a 24 h methanol feed. Signals detected on the immunoblot correspond to 125 µL precipitated culture supernatant. **(C)** Schematic illustrating how peptide chain length affects signal sequence exposure at the ribosome exit tunnel for engagement by SRP or cytosolic HSP70. **(D)** Immunoblot analysis of 3×FLAG secretion using hybrid (tandem) signal peptide constructs. Cultivation and target detection were performed as previously described for **(B)**.

These results prompted consideration of a size-related translocation constraint. During translation, the hydrophobic signal sequence becomes accessible to cytosolic HSP70 chaperones and the signal recognition particle (SRP) only as the nascent chain emerges from the ribosome exit channel (Delic et al., 2013). For very small peptides like 3×FLAG, however, the entire protein chain may not extend beyond the ribosome exit tunnel, limiting signal sequence exposure and thereby impairing co-translational engagement with the translocon or cytosolic chaperones (Wilson and Beckmann, 2011; Zhang et al., 2013) (Figure 2C). To address this, we explored an unconventional strategy of combining two signal peptides in tandem either pre-pro, or pre-pre regions, thereby extending the overall N-terminal region and increasing the window for translocon engagement. Combinations of MFα_pre_-Scw10_pro_ and S30_pre_-Scw10_pro_ abolished secretion, whereas MFα_pre_-Ost1_pre_ and Ost1_pre_-Scw10_pro_ yielded >10-fold higher secretion relative to Ost1_pre_ alone (Figure 2D). However, immunoblot analysis revealed molecular weight shifts for all hybrid constructs, indicating incomplete or aberrant signal sequence processing. As correct N-terminal processing is essential for therapeutic peptide production, these hybrid constructs were not pursued further for direct secretion of single copy peptides, despite their secretion advantage.

### 3.3 Identifying novel effectors of small peptide secretion by random knockout screening

To identify additional bottlenecks beyond ER translocation, we performed random mutagenesis using REMI (restriction enzyme-mediated integration). The Ost1_pre_-3×FLAG secretion strain was transformed with linearized pREMIh, and approximately 3,500 transformants were screened for enhanced secretion using deep-well plate cultivation followed by Anti-FLAG dot blot analysis (Figure 3A).

**FIGURE 3:**
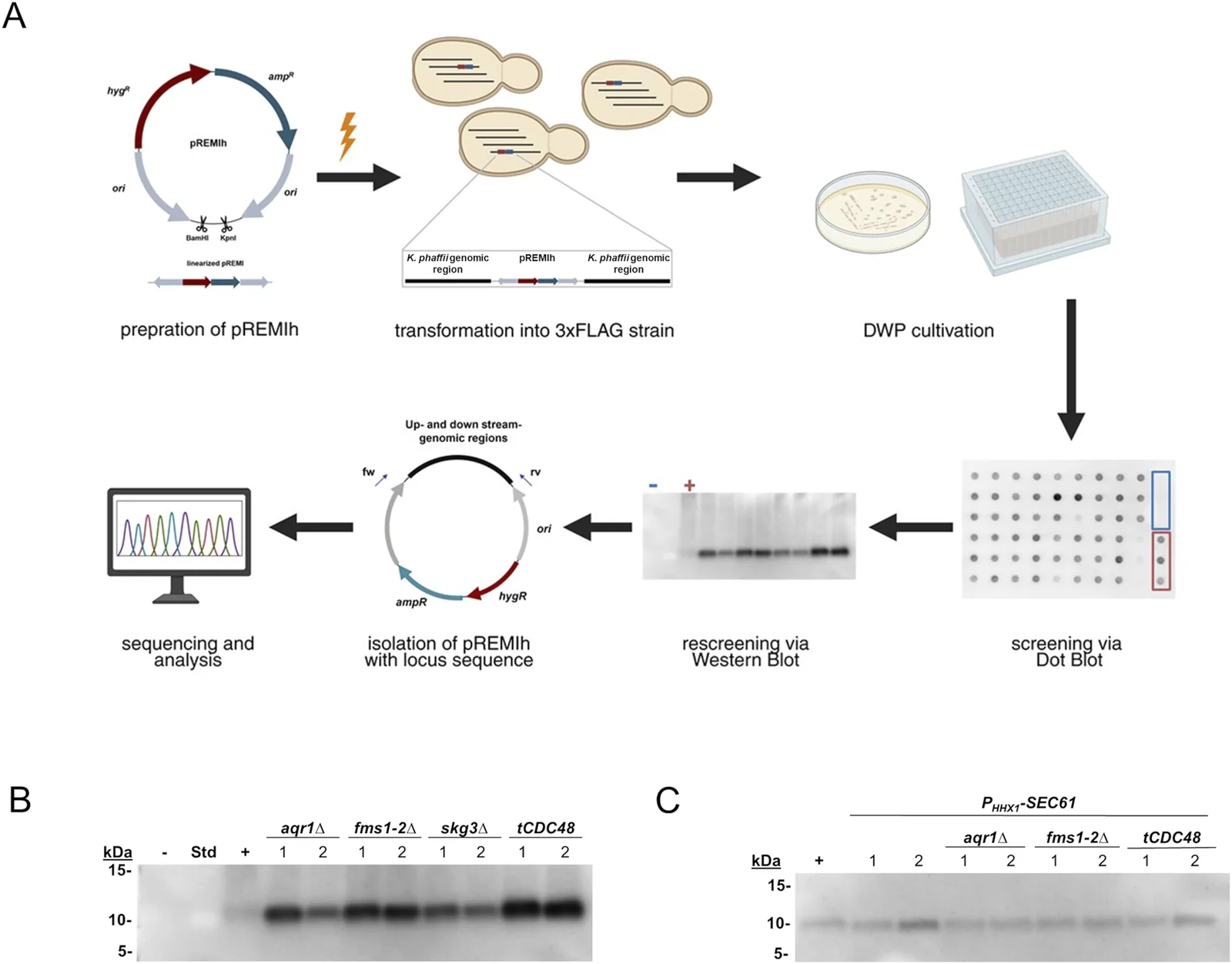
Identification of genetic bottlenecks in 3×FLAG secretion by REMI screening. **(A)** Overview of the REMI-based screening workflow. Random knockout strains were generated by electroporation using the pREMIh plasmid containing a hygromycin resistance cassette for selection in *K. phaffii*. Approximately 3,500 transformants were cultivated in deep-well plates containing BMD for the 60 h batch phase and methanol for 24 h induction. Initial screening of the culture supernatants was done using an Anti-FLAG antibody for dot blot analysis. As controls, the parental 3×FLAG secretion strain using the Ost1_pre_ signal sequence (+) and the wild type CBS7435 his4 strain (−) were included during analysis. Candidate strains were verified by shake flask cultivations and immunoblot. The pREMIh integration loci were identified by isolation of the plasmids from genomic *K. phaffii* DNA and Sanger sequencing remnants of integration site. **(B)** Immunoblot analysis of culture supernatants from strains carrying CRISPR-Cas9-engineered mutations (*AQR1*, *FMS1-2*, *SKG3* frameshifts; *CDC48* truncation). Precipitated culture supernatants (125 µL) of biological duplicates cultivated in shake flasks are shown. After a 60 h batch phase in BMD, production of 3×FLAG was induced by methanol addition for 24 h. **(C)** Immunoblot analysis of strains combining the three best gene knockouts with *SEC61* overexpression by the constitutive *HHX1* promotor (two clones each). +, parental Ost1_pre_-3×FLAG strain; -, wild-type strain. Strains were cultivated as described for **(B)**.

Clones exhibiting elevated secretion signals were rescreened in shake-flask cultures and analyzed by immunoblotting to confirm improved signal intensity and correct apparent molecular weight. The pREMIh plasmids from strains showing reproducible improvements were isolated and sequenced to identify the disrupted genomic loci.

In total, ten gene disruptions associated with enhanced 3×FLAG secretion were identified (Table 1). Four involved disruptions of open reading frames (ORFs), while the remaining cases affected gene truncations or 5′untranslated region (UTR) insertions. Based on reproducibility in a defined genetic background and plausibility of a mechanistic link to secretion, three genes (*AQR1*, *FMS1-2*, and *SKG3*) were selected for CRISPR-Cas9-mediated frameshift mutations, and *CDC48* was selected for targeted C-terminal truncation. All four modifications were reintroduced into the parental Ost1_pre_-3×FLAG strain, and approximately 5- to 9-fold improvements in secretion were observed consistently for biological duplicates (Figure 3B). Combining the three most effective gene modifications with *SEC61* overexpression did not yield additional improvements (Figure 3C). Mass spectrometry of supernatants from the *tCDC48* strain confirmed correct signal sequence processing and an approximate peptide titer of 0.8 mg/L (Supplementary Figure S1).

**TABLE 1:** Genomic loci of pREMIh integration associated with enhanced 3×FLAG secretion.

*K. phaffii* gene	Locus Tag	BLASTp vs. *S. cerevisiae*	Putative function	REMI modification
*AQR1*	BQ9382_C2-0360	8e^−100^/96%	Plasma membrane transporter	Disrupted ORF
*FMS1-2*	BQ9382_C4-0690	2e^−44^/97%	Polyamine oxidase	Disrupted ORF
*SKG3*	BQ9382_C4-1750	5e^−140^/47%	Unknown; bud neck-localized	Disrupted ORF
*CDC48*	BQ9382_C4-0255	0.0/100%	AAA ATPase, protein unfoldase	C-terminal truncation
*NUP120*	BQ9382_C4-5030	6e^−08^/16%	Nuclear pore complex	Disrupted ORF
*YDL206W*	BQ9382_C3-5285	4e^−32^/87%	Cation transporter	5′UTR insertion
*PHS1*	BQ9382_C3-5291	9e^−57^/93%	3-Hydroxyacyl-CoA dehydratase	5′UTR insertion
*DCP1*	BQ9382_C1-0840	9e^−28^/95%	mRNA decapping	5′UTR insertion
*SLC1*	BQ9382_C1-0835	1e^−90^/85%	Acylation of lysophosphatidic acid	5′UTR insertion
*MAK11*	BQ9382_C1-3975	4e^−108^/90%	60S ribosomal subunit biogenesis	C-terminal truncation

BLASTp e-values and query coverage are given for the best *S. cerevisiae* homolog. Putative gene functions were inferred from similarity. Assigned functions/annotations for *SKG3* and *NUP120* are tentative due to low BLASTp, query coverage.

### 3.4 Application of engineering strategies to dQ-Brazzein and CalB secretion

To assess whether the gene modifications identified for 3×FLAG would also enhance secretion of a structurally distinct small peptide, we tested their effects on dQ-brazzein, a well-secreted, disulfide-rich sweet-tasting protein (6.5 kDa). A starting strain carrying a randomly integrated dQ-brazzein expression cassette driven by the *AOX1* promoter and fused to the *S. cerevisiae* Ost1_pre_ signal sequence was constructed, then modified by deletion of *AQR1*, *FMS1-2*, and *SKG3*, or truncation of *CDC48*.

Deletion of *AQR1*, *FMS1-2*, and *SKG3* reproducibly increased dQ-brazzein secretion by approximately two-fold as assessed by SDS-PAGE densitometry (Figure 4A), whereas *CDC48* truncation did not confer the same benefit observed with 3×FLAG. *SEC61* overexpression produced only modest improvement in dQ-brazzein secretion (Figure 4B). Mass spectrometry confirmed the integrity of the secreted dQ-brazzein peptide relative to a synthetic standard (Figure 4C) and showed that disulfide bonds were correctly formed for most of the secreted product. A small fraction of the peptide showed oxidation of cysteines to cysteic acid, indicating one or two missing disulfide bridges and resulting in mass shifts of approximately +98 or +196 Da, respectively. In total, the mass spectrometry data indicated a dQ-brazzein titer of approximately 140 mg/L.

**FIGURE 4:**
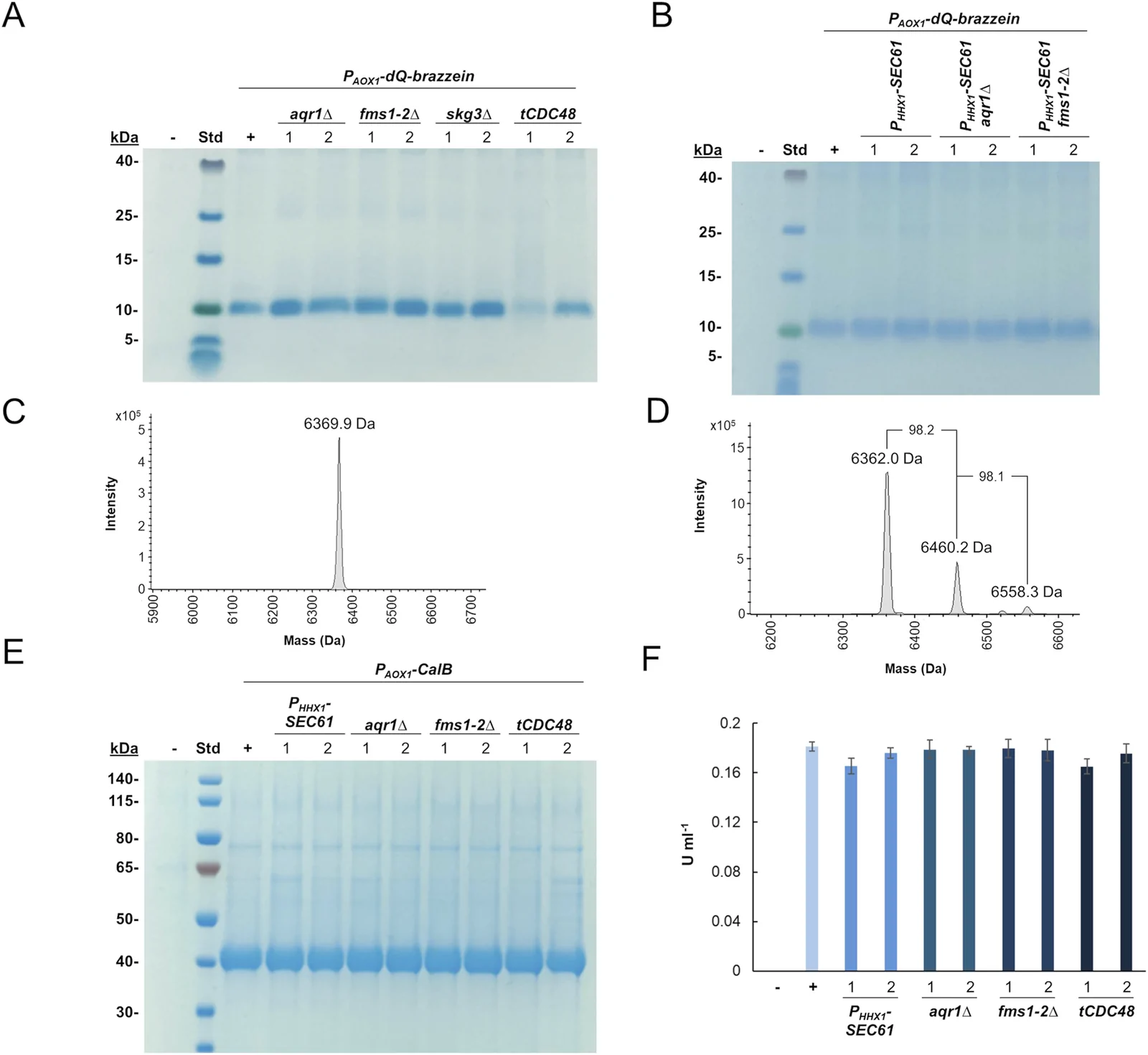
Application of 3×FLAG-derived engineering strategies to dQ-brazzein and CalB secretion. **(A)** SDS-PAGE analysis of dQ-brazzein secretion, mediated by the Ost1_pre_ signal sequence, in strains carrying *AQR1*, *FMS1-2*, or *SKG3* deletions or *CDC48* truncation. *K. phaffii* strains were cultivated for 60 h in BMD containing shake flasks followed by methanol induction for 48 h. Shown are precipitated samples of culture supernatants (125 µL) of biological duplicates for the knockout strains, the parental dQ-brazzein production strain (+) and a BSYBG10 wild type control (−). **(B)** SDS-PAGE of dQ-brazzein secretion in *SEC61* overexpression strains. Two clones are shown for each genetic modification. Cultivations were performed as described previously under **(A)**. +, parental dQ-brazzein secretion strain; -, wild type strain. **(C)** Mass spectrometry analysis of commercial dQ-brazzein standard lacking disulfide bridges. **(D)** Mass spectrometry analysis of secreted dQ-brazzein peptides from the *fms1-2Δ* strain. Integrity of the peptide was confirmed, and partial formation of disulfide bonds was observed. The main peak corresponds to peptide with correctly formed disulfide bridges. The mass shift of approximately +98 corresponds to the full oxidation of two cysteines and one missing disulfide bridge. Peaks correspond to the deconvoluted intact mass of the peptides. **(E)** Extracellular levels and **(F)** enzymatic activity of CalB producing strains with the beneficial genetic modifications for small peptide secretion. Strains were cultivated as described in section **(A)**. Results of the SDS-PAGE and the enzymatic assay with p-nitrophenyl butyrate as substrate, are shown for biological duplicates of the genetic manipulations. As controls the parental CalB secretion strain (+) and the wild type strain BSYBG10 (−) were used.

As a well-secreted reference, *Candida antarctica* lipase B (CalB, ∼33 kDa) was used to probe for a general increase in secretion capacity. CalB secretion was unaffected by any of the gene modifications (Figure 4D), indicating that the observed effects are specific to small peptide secretion and do not reflect a general improvement in secretory capacity.

### 3.5 Application of engineering strategies to therapeutic peptides

To assess whether the engineering strategies identified using 3×FLAG and dQ-brazzein could be transferred to therapeutically relevant targets, we investigated the secretion of the unmodified amino acid sequences of α-melanocyte-stimulating hormone (α-MSH; 13 amino acids, 1.6 kDa) and salmon calcitonin (sCT; 32 amino acids, 3.4 kDa). While α-MSH primarily represents an extreme case of minimal peptide size, salmon calcitonin additionally contains an intramolecular disulfide bridge and has been reported to exhibit aggregation tendencies (Kamgar-Parsi et al., 2017). AlphaFold3 predictions suggested that both peptides adopt partially α-helical conformations, in contrast to the largely unstructured 3×FLAG peptide (Figure 5A). Salmon calcitonin further contains a disulfide bond between Cys1 and Cys7. Expression strains were constructed using the *AOX1* promoter and the *S. cerevisiae* Ost1_pre_ signal sequence, analogous to the expression strategy successfully applied for 3×FLAG and dQ-brazzein.

**FIGURE 5:**
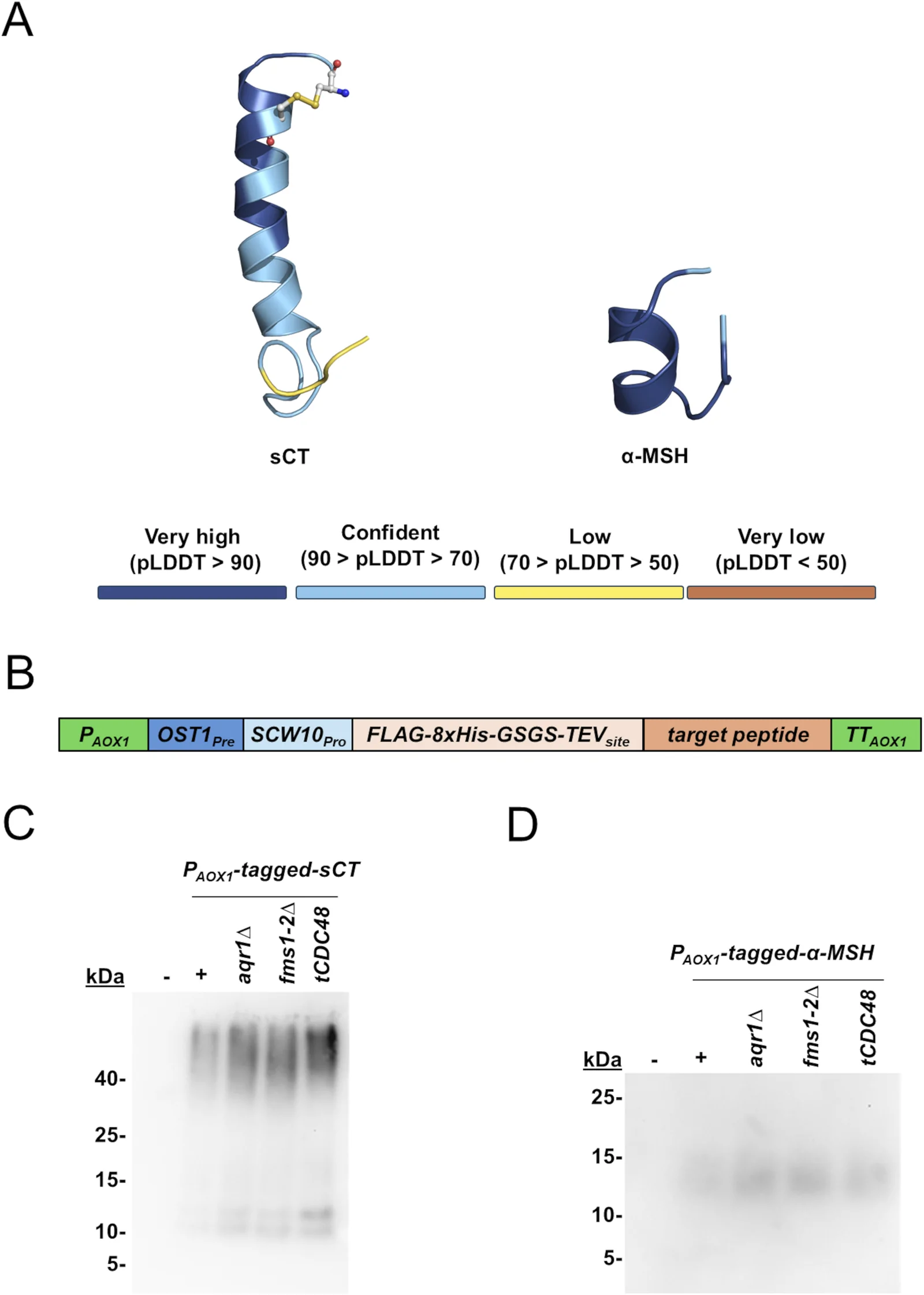
Secretion of challenging therapeutic fusion peptides using extended signal sequences and gene deletions. **(A)** AlphaFold3 structural models including the color coded, predicted local distance difference score (pLDDT) of salmon calcitonin (sCT: 3.4 kDa) and α-melanocyte-stimulating hormone (α-MSH: 1.6 kDa) are shown. Both peptides are predicted to form small α-helical structures with relatively high confidence (pLDDT>70) **(B)** Schematic overview of the expression strategy: Peptides were expressed under the methanol-inducible *AOX1* promoter with the *S. cerevisiae* Ost1_pre_- *K. phaffii* Scw10_pro_-signal sequences combination. N-terminal FLAG-, 8xHis-tags and TEV protease cleavage sites were included to enable detection, purification and release of correctly processed peptides. Mutations (*AQR1*, *FMS1-2*, *CDC48*) were introduced to further enhance secretion of sCT **(C)** and α-MSH **(D)**. Corresponding strains were cultivated in shake flasks containing BMD during a 60 h batch phase. Production of the two pharmaceutic peptides was induced using methanol feed for 24 h. For detection of sCT and α-MSH in the culture supernatant, immunoblots with precipitated supernatant samples corresponding to 125 µL and Anti-FLAG antibody were performed. As controls the parental production strains without additional mutations (+) and the wild type BSYBG10 (−) were included.

However, neither α-MSH nor salmon calcitonin could be detected in culture supernatants by mass spectrometry (Supplementary Figures S2 and S3). Furthermore, none of the engineering strategies that improved 3×FLAG or dQ-brazzein secretion, including *SEC61* overexpression and mutation of *AQR1*, *FMS1-2*, or *CDC48*, resulted in detectable secretion of either therapeutic peptide. These findings indicate either that the limitations associated with secretion of extremely small therapeutic peptides cannot be overcome solely by improving ER translocation or eliminating the genetic bottlenecks identified in the REMI screen or limitations in the MS analysis due to unpredicted target mass changes, for example by peptide aggregation.

We therefore revisited the hybrid signal sequence strategy described above (Figure 2D). Peptides were expressed using an Ost1_pre_-Scw10_pro_ fusion signal sequence and equipped with N-terminal FLAG and 8×His tags separated from the peptide by a TEV protease cleavage site (Figure 5B). This design increased the overall size of the secretory precursor, enabled immunological detection, and allowed generation of the native peptide following TEV cleavage. Using this approach, both peptides became detectable in culture supernatants by immunoblot analysis (Figures 5C,D). Mutations of *AQR1*, *FMS1-2*, and *CDC48* further increased secretion levels of both constructs. For salmon calcitonin, secretion was accompanied by the appearance of a high-molecular-weight smear above 40 kDa, suggesting extensive peptide aggregation during secretion or cultivation (Figure 5C). In contrast, α-MSH predominantly appeared as a diffuse low-molecular-weight signal that increased upon introduction of the beneficial gene deletions (Figure 5D).

To verify peptide identity, enriched culture supernatants were analyzed by mass spectrometry before and after TEV protease cleavage. Due to the low abundance and complexity of the samples, α-MSH could only be detected following TEV cleavage, yielding an estimated concentration of approximately 0.2 μg/L (Supplementary Figure S3). Salmon calcitonin could not be identified by mass spectrometry, likely due to its strong aggregation behavior and low abundance. A comparative overview of all engineering strategies and their effects on peptide production is provided in Table 2.

**TABLE 2:** Overview of engineering strategies and their indicated influence on small peptide production.

Target peptide	Signal sequence	Strain engineering	Relative densitometric quantification	Absolute quantification via MS
3×FLAG	Ost1_pre_	WT	∼100%	​
​	Ost1_pre_	*P*_*HHX1*_ *-SEC61*	∼200%	​
​	Ost1_pre_	*P*_*HHX1*_ *-SEC62*	∼100%	​
​	Ost1_pre_	*P*_*HHX1*_ *-SEC63*	<100%	​
​	Ost1_pre_	*P*_*HHX1*_ *-KAR2*	<100%	​
​	Ost1_pre_	*SEC62-P*_*HHX1*_ *-SEC61*	∼200%	​
​	MFα_pre_-Ost1_pre_	WT	∼800%	​
​	Ost1_pre_-Scw10_pro_	WT	∼1600%	​
​	Ost1_pre_	*aqr1Δ*	∼600%	​
​	Ost1_pre_	*fms1-2Δ*	∼600%	​
​	Ost1_pre_	*skg3Δ*	∼500%	​
​	Ost1_pre_	*tCDC48*	∼900%	0.8 mg/L
dQ-brazzein	Ost1_pre_	WT	∼100%	​
​	Ost1_pre_	*aqr1Δ*	∼200%	​
​	Ost1_pre_	*fms1-2Δ*	∼200%	140 mg/L
​	Ost1_pre_	*skg3Δ*	∼200%	​
​	Ost1_pre_	*tCDC48*	∼100%	​
Linker-sCT	Ost1_pre_-Scw10_pro_	WT	∼100%	​
​	Ost1_pre_-Scw10_pro_	*aqr1Δ*	∼200%	​
​	Ost1_pre_-Scw10_pro_	*fms1-2Δ*	∼200%	​
​	Ost1_pre_-Scw10_pro_	*tCDC48*	∼300%	​
Linker-α-MSH	Ost1_pre_-Scw10_pro_	WT	∼100%	​
​	Ost1_pre_-Scw10_pro_	*aqr1Δ*	∼200%	​
​	Ost1_pre_-Scw10_pro_	*fms1-2Δ*	∼200%	0.2 μg/L
​	Ost1_pre_-Scw10_pro_	*tCDC48*	∼100%	​

Relative quantification is shown relative to the respective parental production strain, together with absolute quantification by HPLC–MS for the best-performing strain of each peptide. Relative quantification of dQ-brazzein was based on Coomassie-stained SDS-PAGE and densitometric analysis of two biological replicates ( n=2 ). The remaining peptides were analyzed by immunoblotting using two biological replicates ( n=2 ).

## 4 Discussion

To date, very few recombinant peptides smaller than 35 amino acids have been secreted using *K. phaffii*. Attempts to produce therapeutically promising antimicrobial peptides such as thanatin and apidaecin, each 18–21 amino acids in length and with strong potential as antibiotic alternatives, have yielded only low extracellular concentrations (Chen et al., 2017; Pipiya et al., 2023). By using 3×FLAG as a model peptide, this study highlights the specific limitations that impede effective secretion of such small proteins in *K. phaffii* and provides a systematic evaluation of engineering strategies for overcoming them. Multiple distinct bottlenecks were identified at the level of signal sequence selection, ER translocation efficiency, and broader cellular processes that collectively constrain the secretion of very small peptides.

A key finding is the critical dependence of small peptide secretion on signal sequence identity. Of the five signal sequences evaluated, only the *S. cerevisiae* Ost1_pre_ signal supported detectable secretion of the 3×FLAG peptide, a result far more extreme than the modest differences typically reported between signal sequences for larger recombinant proteins. This strongly argues that signal sequence selection is not interchangeable for very small peptides. The superior performance of Ost1_pre_ is consistent with its engagement of the co-translational SRP pathway, which may offer a decisive advantage for peptides prone to rapid cytosolic degradation when routed through the post-translational pathway. The strict size dependency of secretion, with extracellular levels declining continuously with each truncation step, suggests that translocation becomes less efficient when the nascent chain cannot fully emerge from the ribosome exit tunnel, potentially limiting signal-sequence recognition by SRP or cytosolic chaperones (Wilson and Beckmann, 2011; Zhang et al., 2013). Notably, intracellular levels declined in parallel with extracellular levels, indicating that limitations arise not only at the translocation step but also at earlier points, potentially at the level of mRNA stability, translation efficiency, or intracellular peptide stability.

Beyond secretion efficiency, correct signal sequence processing is equally critical, particularly for therapeutic applications where a single erroneous N-terminal residue can compromise biological activity or hinder regulatory acceptance. As confirmed by intact mass spectrometry, the *S. cerevisiae* Ost1_pre_ signal achieved precise and complete processing of the 3×FLAG N-terminus. This reliability is especially relevant given the well-documented tendency of the MFα_prepro_ signal to leave heterogeneous N-termini due to incomplete Ste13-mediated removal of its EAEA spacer (Merkaš et al., 2025; Merkaš et al., 2026). For small peptides where the correctly processed product comprises only a handful of amino acids, such processing fidelity is non-negotiable.

The improvement in secretion achieved by *SEC61* overexpression implicates ER translocation capacity as a relevant constraint on 3×FLAG secretion. In contrast, overexpression of *SEC62*, *SEC63*, or the ER luminal chaperone *KAR2* did not consistently improve secretion, arguing against a general chaperone or import factor limitation. This is noteworthy given that small mammalian proteins have been shown to be efficiently secreted via a Sec62-dependent post-translational pathway (Lakkaraju et al., 2012); the lack of a comparable effect in the present *K. phaffii* context may indicate that Ost1_pre_-directed peptides are committed to the co-translational route and therefore bypass Sec62-dependent import entirely. The absence of *SEC62*, *SEC63*, and *KAR2* effects also suggests that luminal chaperone availability and auxiliary translocation factors are not rate-limiting for this small, largely unstructured cargo. The tandem hybrid signal sequence approach, in which combining Ost1_pre_-Scw10_pro_ extended the N-terminal region and produced >10-fold higher secretion relative to Ost1_pre_ alone, corroborates the view that a longer leader increases the window for translocon engagement. However, as immunoblot analysis revealed molecular weight shifts for both hybrid constructs indicating aberrant processing, likely due to problematic engagement with the signal peptide peptidase for the two tandem pre-peptides or the yet unknown proteases necessary for Scw10_pro_ removal.

The REMI screen identified four genetic determinants. *AQR1*, *FMS1-2*, *SKG3*, and *CDC48*, that individually enhanced 3×FLAG secretion by 5- to 9-fold, representing the largest improvements achieved in this study. The function assignments based on sequence similarity, for each is informative. *AQR1* encodes a plasma membrane multidrug resistance-type transporter of the DHA1 family, which reportedly mediate efflux of amino acids including glutamate, aspartate, and alanine (Velasco et al., 2004; Kapetanakis et al., 2021). Its disruption may elevate intracellular amino acid pools in ways that stimulate synthesis or stability of the short 3×FLAG peptide, or may perturb membrane homeostasis in ways that indirectly benefit the secretory pathway. *FMS1-2* encodes a polyamine oxidase involved in the conversion of spermine to spermidine, the essential polyamine precursor for the hypusine modification of eIF5A, which specifically facilitates peptide bond formation at challenging elongation contexts (Chattopadhyay et al., 2003; Dever et al., 2014). Altered polyamine metabolism through *FMS1-2* disruption may therefore shift elongation kinetics in ways that are particularly consequential for small, rapidly synthesized peptides. *CDC48* encodes a conserved AAA-ATPase that extracts ubiquitylated, misfolded secretory proteins from the ER membrane by recruiting the essential co-factors Ufd1/Npl4, playing a central role in ERAD (Stolz et al., 2011; Böhm et al., 2011). The C-terminal truncation identified here impairs the reported co-factor recruitment mechanism, which thereby might reduce degradation of successfully translocated 3×FLAG peptide in the ER lumen. The largely unstructured conformation of 3×FLAG, as predicted by AlphaFold3 (Abramson et al., 2024), may render it particularly vulnerable to ERAD surveillance, which would explain the dramatic impact of Cdc48 perturbation for this specific cargo but the underlying mechanisms for this correlative observation need to be further elucidated. The function of *SKG3*, a bud neck-localized protein, remains poorly characterized and is an intriguing subject for future investigation. The comparably low BLASTp query coverage (47%) further questions if the *K. phaffii* gene is a real homolog to the *S. cerevisiae SKG3*. Overall, no overt growth defects were observed during cultivation for the engineering strategies identified through REMI screening; however, subtle differences in growth rates or final cell densities cannot be excluded at this stage.

Importantly, none of these four targets were identified in comparable screens performed with larger reporter proteins in *K. phaffii* (Ito et al., 2022; Larsen et al., 2013), confirming that small peptide secretion is governed by a partially distinct set of cellular constraints invisible when conventional-sized cargoes are used. The observation that combining the best gene knockouts with *SEC61* overexpression yielded no additional improvement further suggests that the two strategies address independent, parallel rate-limiting steps, an important conceptual guide for future strain engineering.

The partial transferability of the engineering strategies to dQ-brazzein, α-MSH-, and salmon calcitonin-fusion peptides reveals cargo-dependent bottleneck profiles. These results demonstrate that 3×FLAG should be regarded as a practical discovery and screening tool rather than a universal proxy for all peptide classes. While *AQR1*, *FMS1-2*, and *SKG3* deletions consistently improved dQ-brazzein secretion by up to ∼100%, *CDC48* truncation provided no benefit for this structurally compact, disulfide-stabilized peptide, in marked contrast to its dramatic effect on the unstructured 3×FLAG (Caldwell et al., 1998; Kohmura et al., 1996). This contrast supports the model that ERAD-mediated degradation is a dominant bottleneck specifically for unstructured peptides, consistent with the well-established role of Cdc48 in clearing conformationally challenged ER substrates (Stolz et al., 2011; Böhm et al., 2011). It is also worth noting that recent systematic engineering of *K. phaffii* for brazzein production achieved titers of up to 640 mg/L in a bioreactor setting (Rong et al., 2025), approximately 4–5 times higher than the ∼140 mg/L observed here. Differences in expression system design, including the use of the *K. phaffii* S30 signal sequence and co-overexpression of protein disulfide isomerase by Rong et al. (2025) compared to the *S. cerevisiae* Ost1_pre_ signal sequence used in the present study, may contribute to this difference, yet increased brazzein titers *per se* were not the focus of this study.

As exemplified by recent advances in brazzein production, *K. phaffii* is a robust and scalable alternative to plant- and mammalian-cell production systems for disulfide-rich peptides. Its eukaryotic secretory pathway can support oxidative folding and selected post-translational modifications, while secretion into the culture medium can simplify downstream recovery. These features make *K. phaffii* an attractive platform for industrial peptide production, particularly where bacterial or cell-free systems do not provide the required folding or processing capacity (Barone et al., 2023; Taghizadeh et al., 2025; Hashemi et al., 2021).

The results obtained with the α-MSH and salmon calcitonin fusion constructs further emphasize that secretion limitations are strongly cargo-dependent and cannot be predicted from peptide size or therapeutic relevance alone. Both peptides showed poor initial detectability as direct secretory products, requiring the use of the Ost1_pre_–Scw10_pro_ leader and an N-terminal FLAG–8×His–TEV fusion. This design increased the size of the secretory precursor, enabled immunological detection, and may have partially alleviated the size-associated translocation limitation observed for 3×FLAG. Introduction of *AQR1*, *FMS1-2*, or *CDC48* mutations increased immunoblot signal intensity for the fusion constructs, indicating that some engineering targets identified using 3×FLAG can also be beneficial in these distinct expression contexts. However, these observations cannot be used to quantify improvements in mature peptide secretion: α-MSH was detected by mass spectrometry only after TEV cleavage and at a very low estimated concentration (∼0.2 μg/L), whereas salmon calcitonin could not be assigned unambiguously by mass spectrometry.

For salmon calcitonin, the prominent high-molecular-weight smear observed on immunoblots, together with its reported propensity to form amyloid-like fibrillar aggregates under near-physiological conditions, suggests that aggregation or aggregation-associated heterogeneity may complicate secretion and analytical detection (Kamgar-Parsi et al., 2017). Future optimization of such targets should therefore combine the translocation- and ERAD-related interventions described here with peptide-specific strategies, including the evaluation of alternative fusion partners, signal sequences, cultivation conditions, and, where appropriate, chaperones that support folding or limit aggregation.

In addition, mature bioactive α-MSH and salmon calcitonin require post-translational modifications, including C-terminal amidation and N-terminal acetylation, which are not natively introduced by *K. phaffii*. These modifications would need to be incorporated through host engineering or downstream processing for industrial production, analogous to the post-production palmitoylation applied to liraglutide precursor peptides produced in *S. cerevisiae* or *E. coli* (Cheng et al., 2017). Because these modifications can affect peptide stability, their absence during heterologous expression may contribute to the low recovery observed here, although this was not directly tested.

In this study, methanol-inducible expression via the *AOX1* promoter was used throughout, integrating the production process with a carbon capture and utilization approach. Although methanol is classified as a toxic and flammable substrate, a well-recognized challenge for its use in pharmaceutical and food-grade bioprocesses, it is increasingly regarded as a strategically important platform chemical in the context of carbon capture and utilization, as its synthesis from CO_2_ and renewable hydrogen enables direct fixation of atmospheric or industrial carbon into a versatile feedstock (Barone et al., 2023; Kleiber et al., 2021). Importantly, no difference in 3×FLAG secretion was observed between cultivations using CO_2_-derived and commercially available fossil-derived methanol, demonstrating that sustainable methanol can serve as a direct drop-in replacement without compromising recombinant peptide production.

Taken together, this work demonstrates that rational engineering of the ER translocon and targeted disruption of genes involved in peptide degradation, polyamine metabolism, and membrane transport can substantially improve small peptide secretion in *K. phaffii*. The underlying mechanisms should now be resolved using approaches that directly probe translation, ER targeting, and translocation, such as pulse-chase radiolabeling, protease-protection assays, and SRP co-immunoprecipitation. The 3×FLAG model system provides a practical and scalable platform for discovery-oriented screening; however, the identified engineering targets require validation for each target peptide before application in therapeutic or commercial production settings. This limited transferability likely reflects the unusual physicochemical properties of 3×FLAG, including its strong negative charge and largely unstructured character, relative to the structurally and chemically diverse peptides examined here. Future studies should therefore combine the general engineering strategies identified in this work with peptide-specific optimization of codon usage, leader and fusion-partner design, processing strategies, and, where relevant, Golgi-level modifications.

A persistent challenge throughout this work, and one that deserves explicit acknowledgment as a field-wide bottleneck, is the analytical difficulty of detecting, quantifying, and characterizing peptides in this size range. Small peptides are easily lost during downstream processing, pass through standard ultrafiltration membranes, adsorb to surfaces, and are generally elusive on Coomassie-stained SDS-PAGE gels. Mass spectrometry is often essential to confirm correct processing and determine titers, making quality control laborious and inaccessible for routine high-throughput screening. The 3×FLAG model partially addresses this limitation by enabling antibody-based detection; however, equivalent analytical tools are not generally available for newly investigated target peptides. Moreover, the use of SDS-PAGE and immunoblotting limited throughput, allowing only biological duplicates (n = 2) of each strain to be examined and thereby making the resulting secretion quantification primarily indicative. Development of robust, versatile, and high-throughput methods for small-peptide detection and quantification will therefore be essential to increase screening throughput, data comparability, replicate numbers and strain-development workflows.

## Data Availability

The original contributions presented in the study are included in the article/Supplementary Material, further inquiries can be directed to the corresponding author.
