# Supplementary material for: Engineering small peptide secretion in Komagataella phaffii
Source: Front Bioeng Biotechnol. 2026 Sep 16;14:1929744. doi: 10.3389/fbioe.2026.1929744 (PMC13623913; doi:10.3389/fbioe.2026.1929744)
Supplement: Supplementary file 1 [file Supplementaryfile1.docx]

Supplementary Material

**Supplementary Table S1**: Plasmids used in this study.

| **Plasmid** | **Description** | **Application** | **Source** |
| --- | --- | --- | --- |
| pPpT4α | pUC-ori, P_AOX1-Syn_, MFalpha, AOX1_TT_-Syn, P_ILV5_,  P_EM72_, Zeo^R^, AOD_TT_ | vector backbone | (Näätsaari et al., 2012) |
| pPpT4-OST1-3xF | pUC ori, P_ILV5_, P_EM72_, Zeo^R^, AOD_TT_, P_AOX1-Syn_  *OST1-SS*, 3xFLAG, AOX1_TT_-Syn | 3xFLAG secretion | This study |
| pPpT4 MFα -3xF | pUC ori, P_ILV5_, P_EM72_, Zeo^R^, AOD_TT_, P_AOX1-Syn_  *MFα-SS*, 3xFLAG, AOX1_TT_-Syn | 3xFLAG secretion | This study |
| pPpT4 S30-3xF | pUC ori, P_ILV5_, P_EM72_, Zeo^R^, AOD_TT_, P_AOX1-Syn_  *S30_pre_-SS*, 3xFLAG, AOX1_TT_-Syn | 3xFLAG secretion | This study |
| pPpT4 Exp1-3xF | pUC ori, P_ILV5_, P_EM72_, Zeo^R^, AOD_TT_, P_AOX1-Syn_  *EXP1-SS*, 3xFLAG, AOX1_TT_-Syn | 3xFLAG secretion | This study |
| pPpT4-Scw10_prepro_-3xF | pUC ori, P_ILV5_, P_EM72_, Zeo^R^, AOD_TT_, P_AOX1-Syn_  *SCW10_prepro_-SS*, 3xFLAG, AOX1_TT_-Syn | 3xFLAG secretion | This study |
| pPpT4-OST1-3xF-M1 | pUC ori, P_ILV5_, P_EM72_, Zeo^R^, AOD_TT_, P_AOX1-Syn_  *OST1-SS*, 3xFLAG-M1, AOX1_TT_-Syn | 3xFLAG secretion | This study |
| pPpT4-OST1-3xF-M2 | pUC ori, P_ILV5_, P_EM72_, Zeo^R^, AOD_TT_, P_AOX1-Syn_  *OST1-SS*, 3xFLAG-M2, AOX1_TT_-Syn | 3xFLAG secretion | This study |
| pPpT4-OST1-3xF-M3 | pUC ori, P_ILV5_, P_EM72_, Zeo^R^, AOD_TT_, P_AOX1-Syn_  *OST1-SS*, 3xFLAG-M3, AOX1_TT_-Syn | 3xFLAG secretion | This study |
| pPpT4-OST1-3xF-M4 | pUC ori, P_ILV5_, P_EM72_, Zeo^R^, AOD_TT_, P_AOX1-Syn_  *OST1-SS*, 3xFLAG-M4, AOX1_TT_-Syn | 3xFLAG secretion | This study |
| pPpT4-OST1-3xF-M5 | pUC ori, P_ILV5_, P_EM72_, Zeo^R^, AOD_TT_, P_AOX1-Syn_  *OST1-SS*, 3xFLAG-M5, AOX1_TT_-Syn | 3xFLAG secretion | This study |
| pPpT4-OST1-3xF-M6 | pUC ori, P_ILV5_, P_EM72_, Zeo^R^, AOD_TT_, P_AOX1-Syn_  *OST1-SS*, 3xFLAG-M6, AOX1_TT_-Syn | 3xFLAG secretion | This study |
| pPpT4-OST1-3xF-M7 | pUC ori, P_ILV5_, P_EM72_, Zeo^R^, AOD_TT_, P_AOX1-Syn_  *OST1-SS*, 3xFLAG-M7, AOX1_TT_-Syn | 3xFLAG secretion | This study |
| pPpT4 MFα_pre_-Scw10_pro_-3xF | pUC ori, P_ILV5_, P_EM72_, Zeo^R^, AOD_TT_, P_AOX1-Syn_  *MFα_pre_-SCW10_pro_ -SS*, 3xFLAG, AOX1_TT_-Syn | 3xFLAG secretion | This study |
| pPpT4 MFα_pre_-Ost1-3xF | pUC ori, P_ILV5_, P_EM72_, Zeo^R^, AOD_TT_, P_AOX1-Syn_  *MFα_pre_-OST1-SS*, 3xFLAG, AOX1_TT_-Syn | 3xFLAG secretion | This study |
| pPpT4 Ost1-Scw10_pro_-3xF | pUC ori, P_ILV5_, P_EM72_, Zeo^R^, AOD_TT_, P_AOX1-Syn_  *OST1-SCW10_pro_-SS*, 3xFLAG, AOX1_TT_-Syn | 3xFLAG secretion | This study |
| pPpT4 S30-Scw10_pro_-3xF | pUC ori, P_ILV5_, P_EM72_, Zeo^R^, AOD_TT_, P_AOX1-Syn_  *S30-SCW10_pro_-SS*, 3xFLAG, AOX1_TT_-Syn | 3xFLAG secretion | This study |
| pPpT4 S30-Ost1-3xF | pUC ori, P_ILV5_, P_EM72_, Zeo^R^, AOD_TT_, P_AOX1-Syn_  *S30-OST1-SS*, 3xFLAG, AOX1_TT_-Syn | 3xFLAG secretion | This study |
| pREMI-ZA | 2x colE1 ori, P_TEF1_, P_EM7_, Zeo^R^, Amp^R^ | template for plasmid construction of pREMI-HYG | Addgene #183767, (Ito *et al.*, 2022) |
| pREMIh | 2x colE1 ori, P_TEF1_, P_EM7_, Hyg^R^, TEF_TT_, Amp^R^ | random knockout screen | This study |
| A179-HYG | *P_ILV5_, P_EM72_, Hyg^R^, AOD_TT_, pARS1, pUC ori, P_HTX1,_ HsCas9 AOX1TT, sgRNA* | template for CRISPR-plasmid generation | (Lehmayer *et al.* 2022) |
| CRISPR-AQR1 | *P_ILV5_, P_EM72_, Hyg^R^, AOD_TT_, pARS1, pUC ori, P_HTX1,_ HsCas9 AOX1TT, sgRNA*, *AQR*-protospacer | *AQR1* knockout | This study |
| CRISPR-FSM1 | *P_ILV5_, P_EM72_, Hyg^R^, AOD_TT_, pARS1, pUC ori, P_HTX1,_ HsCas9 AOX1TT, sgRNA* *FMS1-2*-protospacer | *FMS1-2* knockout | This study |
| CRISPR-SKG | *P_ILV5_, P_EM72_, Hyg^R^, AOD_TT_, pARS1, pUC ori, P_HTX1,_ HsCas9 AOX1TT, sgRNA,* *SKG3*-protospacer | *SKG3* knockout | This study |
| CRISPR-CDC48 | *P_ILV5_, P_EM72_, Hyg^R^, AOD_TT_, pARS1, pUC ori, P_HTX1,_ HsCas9 AOX1TT, sgRNA*, *CDC48*-protospacer | C-terminal truncation of *CDC48* | This study |
| pPpT4-OST1-braz | pUC ori, P_ILV5_, P_EM72_, Zeo^R^, AOD_TT_, P_AOX1-Syn_  *OST1-SS, dQ-brazzein, AOX1_TT_-Syn* | dQ-brazzein secretion | This study |
| pPpT4α-CalB | pUC ori, P_ILV5_, P_EM72_, Zeo^R^, AOD_TT_, P_AOX1-Syn_  *MFα-SS*, *CALB*, AOX1_TT_-Syn | CalB secretion | (Vogl *et al.*, 2016) |
| pPpT4-OST1-Scw10_pro_-sCT | *pUC ori, P_ILV5_, P_EM72_, Zeo^R^, AOD_TT_, P_AOX1-Syn_*  *OST1-SCW10_pro_-SS, FLAG-6xHis-TEV, sCT, AOX1_TT_-Syn* | Secretion of salmon calcitonin with a TEV-cleavable N-terminal linker | This study |
| pPpT4-OST1-Scw10_pro_-αMSH | *pUC ori, P_ILV5_, P_EM72_, Zeo^R^, AOD_TT_, P_AOX1-Syn_*  *OST1-SCW10_pro_-SS, FLAG-6xHis-TEV, αMSH, AOX1_TT_-Syn* | Secretion of αMSH with a TEV-cleavable N-terminal linker | This study |

**Supplementary Table S2:** Synthesized DNA fragments used during strain construction. For CRISPR/Cas9 plasmids underlined letters indicate the protospacer and hammer head sequences. For the gB fragments upper case letters indicate ORF, underlined bases indicate the secreted target peptide.

| **Primer** | **Sequence** | **Application** |
| --- | --- | --- |
| gB-Ost1-3xFLAG | acgacaacttgagaagatcaaaaaacaactaattattgaaagaattcATGAGGCAGGTTTGGTTCTCTTGGATTGTGGGATTGTTCCTATGTTTTTTCAACGTGTCTTCTGCTATGGATTATAAGGATCATGACGGTGATTACAAAGACCATGATATTGACTACAAAGATGACGATGACAAGTAAgcggccgctcaagaggatgtcagaatgccatttgcctgagagatgc | 3xFLAG expression plasmid with Ost1 signal sequence |
| gB-MFα-3xF | acgacaacttgagaagatcaaaaaacaactaattattgaaagaattcATGAGATTCCCATCTATTTTCACCGCTGTCTTGTTCGCTGCCTCCTCTGCATTGGCTGCCCCTGTTAACACTACCACTGAAGACGAGACTGCTCAAATTCCAGCTGAAGCAGTTATCGGTTACTCTGACCTTGAGGGTGATTTCGACGTCGCTGTTTTGCCTTTCTCTAACTCCACTAACAACGGTTTGTTGTTCATTAACACCACTATCGCTTCCATTGCTGCTAAGGAAGAGGGTGTCTCTCTCGAGAAGAGAATGGATTATAAGGATCATGACGGTGATTACAAAGACCATGATATTGACTACAAAGATGACGATGACAAGTAAgcggccgctcaagaggatgtcagaatgccatttgcctgagagatgc | 3xFLAG expression plasmid with mating factor alpha signal sequence |
| gB-S30-3xF | acgacaacttgagaagatcaaaaaacaactaattattgaaagaattcATGAAGTTCGCAATTTCAACACTTCTTATTATCCTACAGGCTGCCGCTGTTTTTGCTGCCATGGATTATAAGGATCATGACGGTGATTACAAAGACCATGATATTGACTACAAAGATGACGATGACAAGTAAgcggccgctcaagaggatgtcagaatgccatttgcctgagagatgc | 3xFLAG expression plasmid with S30 signal sequence |
| pPpT4 Exp1-3xF | AcgacaacttgagaagatcaaaaaacaactaattattgaaagaattcATGAAGCTCTCCACCAATTTGATTCTAGCTATTGCAGCAGCTTCCGCCGTTGTCTCAGCTATGGATTATAAGGATCATGACGGTGATTACAAAGACCATGATATTGACTACAAAGATGACGATGACAAGTAAgcggccgctcaagaggatgtcagaatgccatttgcctgagagatgc | 3xFLAG expression plasmid with Exp1 signal sequence |
| gB-Scw10_prepro_-3xF | acgacaacttgagaagatcaaaaaacaactaattattgaaagaattcATGCAAGTTAAATCTATCGTTAACCTACTGTTGGCATGTTCGTTGGCCGTGGCCAGACCTTTGGAGCACGCCCACCATCAACATGACAAGAGAATGGATTATAAGGATCATGACGGTGATTACAAAGACCATGATATTGACTACAAAGATGACGATGACAAGTAAgcggccgctcaagaggatgtcagaatgccatttgcctgagagatgc | 3xFLAG expression plasmid with Scw10 signal sequence |
| gB-OST1-3xF-M1 | acgacaacttgagaagatcaaaaaacaactaattattgaaagaattcATGAGGCAGGTTTGGTTCTCTTGGATTGTGGGATTGTTCCTATGTTTTTTCAACGTGTCTTCTGCTATGGATAAGGATCATGACGGTGATTACAAAGACCATGATATTGACTACAAAGATGACGATGACAAGTAAgcggccgctcaagaggatgtcagaatgccatttgcctgagagatgc | expression plasmid for 3xFLAG truncation |
| gB-OST1-3xF-M2 | acgacaacttgagaagatcaaaaaacaactaattattgaaagaattcATGAGGCAGGTTTGGTTCTCTTGGATTGTGGGATTGTTCCTATGTTTTTTCAACGTGTCTTCTGCTATGGATGATCATGACGGTGATTACAAAGACCATGATATTGACTACAAAGATGACGATGACAAGTAAgcggccgctcaagaggatgtcagaatgccatttgcctgagagatgc | expression plasmid for 3xFLAG truncation |
| gB-OST1-3xF-M3 | acgacaacttgagaagatcaaaaaacaactaattattgaaagaattcATGAGGCAGGTTTGGTTCTCTTGGATTGTGGGATTGTTCCTATGTTTTTTCAACGTGTCTTCTGCTATGGATCATGACGGTGATTACAAAGACCATGATATTGACTACAAAGATGACGATGACAAGTAAgcggccgctcaagaggatgtcagaatgccatttgcctgagagatgc | expression plasmid for 3xFLAG truncation |
| gB-OST1-3xF-M4 | acgacaacttgagaagatcaaaaaacaactaattattgaaagaattcATGAGGCAGGTTTGGTTCTCTTGGATTGTGGGATTGTTCCTATGTTTTTTCAACGTGTCTTCTGCTATGGATGACGGTGATTACAAAGACCATGATATTGACTACAAAGATGACGATGACAAGTAAgcggccgctcaagaggatgtcagaatgccatttgcctgagagatgc | expression plasmid for 3xFLAG truncation |
| gB-OST1-3xF-M5 | acgacaacttgagaagatcaaaaaacaactaattattgaaagaattcATGAGGCAGGTTTGGTTCTCTTGGATTGTGGGATTGTTCCTATGTTTTTTCAACGTGTCTTCTGCTATGGATGGTGATTACAAAGACCATGATATTGACTACAAAGATGACGATGACAAGTAAgcggccgctcaagaggatgtcagaatgccatttgcctgagagatgc | expression plasmid for 3xFLAG truncation |
| gB-OST1-3xF-M6 | acgacaacttgagaagatcaaaaaacaactaattattgaaagaattcATGAGGCAGGTTTGGTTCTCTTGGATTGTGGGATTGTTCCTATGTTTTTTCAACGTGTCTTCTGCTATGGATGATTACAAAGACCATGATATTGACTACAAAGATGACGATGACAAGTAAgcggccgctcaagaggatgtcagaatgccatttgcctgagagatgc | expression plasmid for 3xFLAG truncation |
| gB--OST1-3xF-M7 | acgacaacttgagaagatcaaaaaacaactaattattgaaagaattcATGAGGCAGGTTTGGTTCTCTTGGATTGTGGGATTGTTCCTATGTTTTTTCAACGTGTCTTCTGCTATGGATTACAAAGACCATGATATTGACTACAAAGATGACGATGACAAGTAAgcggccgctcaagaggatgtcagaatgccatttgcctgagagatgc | expression plasmid for 3xFLAG truncation |
| pCR-SecARG4 fwd | GGATCCtatactgagtttgttaatgatacaataaac | Overexpression of ER translocon components |
| pCR_Sec_Stuffer_rv1 | AAATATATAAAAATTAATTCAACAGTAGCAAAGTATTG | Overexpression of ER translocon components |
| pCR-HHX-fw1 | ttttctttacctggatataaataaaaaaaagg | Overexpression of ER translocon components |
| pCR-HHX-rv2 | gcggccgcgttttatcgatagtagttgagcaataa | Overexpression of ER translocon components |
| pCR_Sec_AOX1TT_fw1 | gcacaaacgaaggtctcac | Overexpression of ER translocon components |
| pCR_Sec_AOX1TT_rv1 | tatttatatccaggtaaagaaaagagctcaacgtttataa tcaagaggatgtcagaatgc | Overexpression of ER translocon components |
| pCR-Sec61_f1 | gctcaactactatcgataaaacgcggccgc atgagcagcttcagagttc | Overexpression of ER translocon components |
| pCR-Sec61_r1 | tgtatcattaacaaactcagtataGGATCC ttacagatcaacgaatccagag | Overexpression of ER translocon components |
| pCR-Sec62_f1 | gctcaactactatcgataaaacgcggccgc atggagcaggttccag | Overexpression of ER translocon components |
| pCR-Sec62_r1 | tgtatcattaacaaactcagtataGGATCC ttattcatcataaacttcttctatggtg | Overexpression of ER translocon components |
| pCR-Sec63_f1 | gctcaactactatcgataaaacgcggccgc ATGGCACGAGTCGAGTATG | Overexpression of ER translocon components |
| pCR-Sec63_r1 | tgtatcattaacaaactcagtataGGATCC CTATTTATCTTCACCCTCATCTTC | Overexpression of ER translocon components |
| pCR-Kar2_f1 | gctcaactactatcgataaaacgcggccgc atgctgtcgttaaaaccatcttg | Overexpression of ER translocon components |
| pCR-Kar2_r1 | tgtatcattaacaaactcagtataGGATCC ctacaactcatcatgatcatagtcatag | Overexpression of ER translocon components |
| pCR_Sec_AOX1TT_rv2 | tcaagaggatgtcagaatgc | Overexpression of ER translocon components |
| pCR-Sec61_f2 | ttataatcaagaggatgtcagaatgccatt ttacagatcaacgaatccagag | Overexpression of ER translocon components |
| pCR-Sec61_r2 | ttatttatatccaggtaaagaaaagagctc atgagcagcttcagagttctag | Overexpression of ER translocon components |
| pCR-Sec62_f2 | gctcaactactatcgataaaacgcggccgc atggagcaggttccagtc | Overexpression of ER translocon components |
| pCR-Sec62_r2 | tgtatcattaacaaactcagtataGGATCC ttattcatcataaacttcttctatggtg | Overexpression of ER translocon components |
| fw_seq_pHHX1 | tgctaacaaggtgagccatc | Overexpression of ER translocon components |
| rev_seq_Arg4TT | ctgcaaataatcgaagaagagaccc | Overexpression of ER translocon components |
| gB-MFα_pre_-Scw10_pro_-3xF | acgacaacttgagaagatcaaaaaacaactaattattgaaagaattcATGAGATTCCCATCTATTTTCACCGCTGTCTTGTTCGCTGCCTCCTCTGCATTGGCTGCCAGACCTTTGGAGCACGCCCACCATCAACATGACAAGAGAATGGATTATAAGGATCATGACGGTGATTACAAAGACCATGATATTGACTACAAAGATGACGATGACAAGTAAgcggccgctcaagaggatgtcagaatgccatttgcctgagagatgc | 3xFLAG expression plasmid with hybrid signal sequence |
| gB-MFα_pre_-Ost1-3xF | acgacaacttgagaagatcaaaaaacaactaattattgaaagaattcATGAGATTCCCATCTATTTTCACCGCTGTCTTGTTCGCTGCCTCCTCTGCATTGGCTGCCAGGCAGGTTTGGTTCTCTTGGATTGTGGGATTGTTCCTATGTTTTTTCAACGTGTCTTCTGCTATGGATTATAAGGATCATGACGGTGATTACAAAGACCATGATATTGACTACAAAGATGACGATGACAAGTAAgcggccgctcaagaggatgtcagaatgccatttgcctgagagatgc | 3xFLAG expression plasmid with hybrid signal sequence |
| gB-Ost1-Scw10pro-3xF | acgacaacttgagaagatcaaaaaacaactaattattgaaagaattcATGAGGCAGGTTTGGTTCTCTTGGATTGTGGGATTGTTCCTATGTTTTTTCAACGTGTCTTCTGCTAGACCTTTGGAGCACGCCCACCATCAACATGACAAGAGAATGGATTATAAGGATCATGACGGTGATTACAAAGACCATGATATTGACTACAAAGATGACGATGACAAGTAAgcggccgctcaagaggatgtcagaatgccatttgcctgagagatgc | 3xFLAG expression plasmid with hybrid signal sequence |
| gB-S30-Scw10pro-3xF | AcgacaacttgagaagatcaaaaaacaactaattattgaaagaattcATGAAGTTCGCAATTTCAACACTTCTTATTATCCTACAGGCTGCCGCTGTTTTTGCTGCCAGACCTTTGGAGCACGCCCACCATCAACATGACAAGAGAATGGATTATAAGGATCATGACGGTGATTACAAAGACCATGATATTGACTACAAAGATGACGATGACAAGTAAgcggccgctcaagaggatgtcagaatgccatttgcctgagagatgc | 3xFLAG expression plasmid with hybrid signal sequence |
| gB-S30-Ost1-3xF | AcgacaacttgagaagatcaaaaaacaactaattattgaaagaattcATGAAGTTCGCAATTTCAACACTTCTTATTATCCTACAGGCTGCCGCTGTTTTTGCTGCCAGGCAGGTTTGGTTCTCTTGGATTGTGGGATTGTTCCTATGTTTTTTCAACGTGTCTTCTGCTATGGATTATAAGGATCATGACGGTGATTACAAAGACCATGATATTGACTACAAAGATGACGATGACAAGTAAgcggccgctcaagaggatgtcagaatgccatttgcctgagagatgc | 3xFLAG expression plasmid with hybrid signal sequence |
| pCR-REMI-HygR_fw1 | tatatcggcatagtataatacgacaaggtgaggaactaaacatgggtaaaaagcctgaac | Construction of pREMIh |
| pCR-REMI-TEFTT_rv1 | cggcatcagagcagattgtactgagagtgcaccatatcgcgttcaatataccag | Construction of pREMIh |
| pSeq-REMI-HygR_fw1 | gcatagcaatctaatctaag | Construction of pREMIh |
| pSeq-REMI_HygR_rv1 | tcggggctggcttaactatg | Construction of pREMIh |
| pSeq-REMI-Amp_fw3 | gatctaggtgaagatcc | Identification of integration loci |
| pSeq-REMI-Amp_fw4 | cagtgagcgcaacgc | Identification of integration loci |
| pSeq-REMI-rv1 | gcctttttacggttcc | Identification of integration loci |
| pSeq-REMI_rv2 | gcatgcgtagaaaagatc | Identification of integration loci |
| pCRISPR-AQR1_f1 | ACGAAACGAGTAAGCTCGTCGAAGAGGGACAAGCCAGAGGGTTTTAGAGCTAGAAATAGC | *AQR1* CRISPR/Cas9 plasmid |
| rev_F1_pCRISPR | GGGCATCACAATCATGGAGC | CRISPR/Cas9 plasmid |
| fw_F2_pCRISPR | CCTCGAGAAAGTCGATGGGG | CRISPR/Cas9 plasmid |
| pCRISPR-AQR1_r1 | GAGCTTACTCGTTTCGTCCTCACGGACTCATCAGGAAGAGTTTGATTTGTTTAGGTAACT | *AQR1* CRISPR/Cas9 plasmid |
| pSeq-AQR1_fw1 | GGAGCATATTATTCCG | Control of *aqr1* knockout |
| pCont-CRISPR-AQR1_fw1 | CCGCCAGACTGTCTG | Control of *aqr1* knockout |
| pCont-CRISPR-AQR1_rv1 | CTTTCTAGTCAAAGCCAAG | Control of *aqr1* knockout |
| pCRISPR-FSM1_f1 | ACGAAACGAGTAAGCTCGTCCCCCAAATCCAATGAGACTGGTTTTAGAGCTAGAAATAGC | *FMS1-2* CRISPR/Cas9 plasmid |
| pCRISPR-FSM1_r1 | GAGCTTACTCGTTTCGTCCTCACGGACTCATCAGCCCCAATTTGATTTGTTTAGGTAACT | *FMS1-2* CRISPR/Cas9 plasmid |
| pSeq-FSM1-2_r1 | GGATGTTAGATCTCG | Control of *fms1-2* knockout |
| pCont-CRISPR-FSM_fw1 | GCACCACCAATCTCAG | Control of *fms1-2* knockout |
| pCont-CRISPR-FSM1_rv1 | GTGGATGTCGGATGGC | Control of *fms1-2* knockout |
| pCRISPR-SKG3_f1 | ACGAAACGAGTAAGCTCGTCCCCACTGCTCTGTAAATTGGGTTTTAGAGCTAGAAATAGC | *SKG3* CRISPR/Cas9 plasmid |
| pCRISPR-SKG3_r1 | GAGCTTACTCGTTTCGTCCTCACGGACTCATCAGCCCACTTTTGATTTGTTTAGGTAACT | *SKG3* CRISPR/Cas9 plasmid |
| pSeq-SKG3_rv1 | CCAATCCCCGTTCTTAGC | Control of *skg3* knockout |
| pCont-CRISPR-SKG3_fw1 | CGGTAAAGTATCCAAAGC | Control of *skg3* knockout |
| pCont-CRISPR-SKG3_rv1 | CGTCTACACTAAAGACTAC | Control of *skg3* knockout |
| pCRISPR-CDC48_f1 | ACGAAACGAGTAAGCTCGTCGAATCCAGTGGGCCCGCTTTGTTTTAGAGCTAGAAATAGC | *CDC48* CRISPR/Cas9 plasmid |
| pCRISPR-CDC48_r1 | GAGCTTACTCGTTTCGTCCTCACGGACTCATCAGGAATCCTTTGATTTGTTTAGGTAACT | *CDC48* CRISPR/Cas9 plasmid |
| pRep-CDC48_f1 | GAGAATCTGCTGGCAATGGCGGTTCTATTGGTGCTGAATCCAGTGGGtaaGCTTTCTAAC | Repair oligo for CDC48 truncation |
| pRep-CDC48_r1 | GAACGCATCAACTATACAAATCGTCATCATCGGGCTCAACGTTAGAAAGCttaCCCACTG | Repair oligo for CDC48 truncation |
| pSeq_CDC48_f1 | ATTGGATCTTCAGGAGATTGCTAAG | Control of *CDC48* truncation |
| pCont_CDC48_f1 | CCAGCTAGGCTGTCCATTC | Control of *CDC48* truncation |
| pCont_CDC48_r1 | CTTCGACCGAAATGTGAACAC | Control of *CDC48* truncation |
| gB-braz | acgacaacttgagaagatcaaaaaacaactaattattgaaacgATGAGGCAGGTTTGGTTCTCTTGGATTGTGGGATTGTTCCTATGTTTTTTCAACGTGTCTTCTGCTGATAAATGTAAAAAGGTTTACGAAAATTATCCAGTTTCTAAGTGTCAATTAGCTAACCAGTGTAACTACGATTGTAAATTGGATAAACATGCTAGATCCGGTGAGTGCTTCTATGACGAAAAGAGAAACTTGCAGTGTATTTGTGATTATTGCGAATATTAAgcggccgctcaagaggatgtcagaatgccatttgcctgagagatgc | dQ-brazzein expression plasmid |
| gB-MSH | acgacaacttgagaagatcaaaaaacaactaattattgaaagaattcATGAGGCAGGTTTGGTTCTCTTGGATTGTGGGATTGTTCCTATGTTTTTTCAACGTGTCTTCTGCTAGACCTTTGGAGCACGCCCACCATCAACATGACAAGAGAatggactacaaagatgacgatgacaagcaccatcaccatcaccatcaccatggttctggatccgaaaacctatatttccaaTCCTACTCTATGGAGCACTTCAGATGGGGAAAACCTGTTTAAgcggccgctcaagaggatgtcagaatgccatttgcctgagagatgc | αMSH expression plasmid containing a N-terminal linker |
| gB-sCT | acgacaacttgagaagatcaaaaaacaactaattattgaaagaattcATGAGGCAGGTTTGGTTCTCTTGGATTGTGGGATTGTTCCTATGTTTTTTCAACGTGTCTTCTGCTAGACCTTTGGAGCACGCCCACCATCAACATGACAAGAGAatggactacaaagatgacgatgacaagcaccatcaccatcaccatcaccatggttctggatccgaaaacctatatttccaaTGTTCCAACCTTTCCACCTGCGTGTTGGGTAAACTAAGTCAGGAACTTCATAAGCTACAAACTTACCCTAGAACAAATACTGGTTCTGGTACGCCTTAAgcggccgctcaagaggatgtcagaatgccatttgcctgagagatgc | sCT expression plasmid containing a N-terminal linker |
| rev_check_AOX1TT | aggagtgggaaataccaag | Sequencing of expression plamids |
| pCont-Paox_f1 | gaaggaagctgccctgtc | Colony PCR for successful integration |
| pCont-Pilv_r1 | atggctcaccactgcaac | Colony PCR for successful integration |

**Supplementary Table S3:** *K. phaffii* strains used during this study.

| **Strain Description** | **Source** |
| --- | --- |
| CBS7435 *his4*Δ | (Näätsaari *et al.*, 2012) |
| CBS7435 *his4*Δ *P_AOX1_-ScOST1-3xFLAG ZEO^R^* | This study |
| CBS7435 *his4*Δ *P_AOX1_-ScMFα-3xFLAG ZEO^R^* | This study |
| CBS7435 *his4*Δ *P_AOX1_-KpS30-3xFLAG ZEO^R^* | This study |
| CBS7435 *his4*Δ *P_AOX1_-KpEXP1-3xFLAG ZEO^R^* | This study |
| CBS7435 *his4*Δ *P_AOX1_-KpSCW10-3xFLAG ZEO^R^* | This study |
| CBS7435 *his4*Δ *P_AOX1_-ScOST1-3xFLAG-M1 ZEO^R^* | This study |
| CBS7435 *his4*Δ *P_AOX1_-ScOST1-3xFLAG-M2 ZEO^R^* | This study |
| CBS7435 *his4*Δ *P_AOX1_-ScOST1-3xFLAG-M3 ZEO^R^* | This study |
| CBS7435 *his4*Δ *P_AOX1_-ScOST1-3xFLAG-M4 ZEO^R^* | This study |
| CBS7435 *his4*Δ *P_AOX1_-ScOST1-3xFLAG-M5 ZEO^R^* | This study |
| CBS7435 *his4*Δ *P_AOX1_-ScOST1-3xFLAG-M6 ZEO^R^* | This study |
| CBS7435 *his4*Δ *P_AOX1_-ScOST1-3xFLAG-M7 ZEO^R^* | This study |
| CBS7435 *his4*Δ *P_AOX1_-ScOST1-3xFLAG ZEO^R^ P_HHX1_-KpSec61 HYG^R^* | This study |
| CBS7435 *his4*Δ *P_AOX1_-ScOST1-3xFLAG ZEO^R^ P_HHX1_-KpSec62 HYG^R^* | This study |
| CBS7435 *his4*Δ *P_AOX1_-ScOST1-3xFLAG ZEO^R^ P_HHX1_-KpSec63 HYG^R^* | This study |
| CBS7435 *his4*Δ *P_AOX1_-ScOST1-3xFLAG ZEO^R^ P_HHX1_-KpKAR2 HYG^R^* | This study |
| CBS7435 *his4*Δ *P_AOX1_-ScOST1-3xFLAG ZEO^R^ KpSEC62-P_HHX1_-KpSEC61 HYG^R^* | This study |
| CBS7435 *his4*Δ *P_AOX1_-ScMFα_pre_-KpSCW10_pro_-3xFLAG ZEO^R^* | This study |
| CBS7435 *his4*Δ *P_AOX1_-ScMFα_pre_-ScOST1-3xFLAG ZEO^R^* | This study |
| CBS7435 *his4*Δ *P_AOX1_-ScOST1-KpSCW10_pro_-3xFLAG ZEO^R^* | This study |
| CBS7435 *his4*Δ *P_AOX1_-KpS30-KpSCW10-3xFLAG ZEO^R^* | This study |
| CBS7435 *his4*Δ *P_AOX1_-KpS30-ScOST1-3xFLAG ZEO^R^* | This study |
| CBS7435 *his4*Δ *P_AOX1_-ScOST1-3xFLAG ZEO^R^ aqr1∆* | This study |
| CBS7435 *his4*Δ *P_AOX1_-ScOST1-3xFLAG ZEO^R^ fms1-2∆* | This study |
| CBS7435 *his4*Δ *P_AOX1_-ScOST1-3xFLAG ZEO^R^ skg3∆* | This study |
| CBS7435 *his4*Δ *P_AOX1_-ScOST1-3xFLAG ZEO^R^ tCDC48* | This study |
| CBS7435 *his4*Δ *P_AOX1_-ScOST1-3xFLAG ZEO^R^ P_HHX1_-KpSec61 HYG^R^ aqr1∆* | This study |
| CBS7435 *his4*Δ *P_AOX1_-ScOST1-3xFLAG ZEO^R^ P_HHX1_-KpSec61 HYG^R^ fms1-2∆* | This study |
| CBS7435 *his4*Δ *P_AOX1_-ScOST1-3xFLAG ZEO^R^ P_HHX1_-KpSec61 HYG^R^ skg3∆* | This study |
| CBS7435 *his4*Δ *P_AOX1_-ScOST1-3xFLAG ZEO^R^ P_HHX1_-KpSec61 HYG^R^ tCDC48* | This study |
| BSYBG10 | bisy GmbH (Hofstätten an der Raab, Austria) |
| BSYBG10 *P_AOX1_-ScOST1-dQ-brazzein ZEO^R^* | This study |
| BSYBG10 *P_AOX1_-ScOST1-dQ-brazzein ZEO^R^ aqr1∆* | This study |
| BSYBG10 *P_AOX1_-ScOST1-dQ-brazzein ZEO^R^ fms1-2∆* | This study |
| BSYBG10 *P_AOX1_-ScOST1-dQ-brazzein ZEO^R^ skg3∆* | This study |
| BSYBG10 *P_AOX1_-ScOST1-dQ-brazzein ZEO^R^ tCDC48* | This study |
| BSYBG10 *P_AOX1_-ScOST1-dQ-brazzein ZEO^R^ P_HHX1_-KpSec61 HYG^R^* | This study |
| BSYBG10 *P_AOX1_-ScOST1-dQ-brazzein ZEO^R^ P_HHX1_-KpSec61 HYG^R^ aqr1∆* | This study |
| BSYBG10 *P_AOX1_-ScOST1-dQ-brazzein ZEO^R^ P_HHX1_-KpSec61 HYG^R^ fms1-2∆* | This study |
| BSYBG10 *P_AOX1_-ScOST1-dQ-brazzein ZEO^R^ P_HHX1_-KpSec61 HYG^R^ skg3∆* | This study |
| BSYBG10 *P_AOX1_-ScOST1-dQ-brazzein ZEO^R^ P_HHX1_-KpSec61 HYG^R^ tCDC48* | This study |
| BSYBG10 *P_AOX1_-ScMFα-CALB ZEO^R^* | This study |
| BSYBG10 *P_AOX1_-ScMFα-CALB ZEO^R^ P_HHX1_-KpSec61 HYG^R^* | This study |
| BSYBG10 *P_AOX1_-ScMFα-CALB ZEO^R^ aqr1∆* | This study |
| BSYBG10 *P_AOX1_-ScMFα-CALB ZEO^R^ fms1-2∆* | This study |
| BSYBG10 *P_AOX1_-ScMFα-CALB ZEO^R^ tCDC48* | This study |
| BSYBG10 *P_AOX1_-ScOST1-KpSCW10_pro_-FLAG-8xHis-GSGS-TEV_site_-sCT ZEO^R^* | This study |
| BSYBG10 *P_AOX1_-ScOST1-KpSCW10_pro_-FLAG-8xHis-GSGS-TEV_site_-sCT ZEO^R^ aqr1∆* | This study |
| BSYBG10 *P_AOX1_-ScOST1-KpSCW10_pro_-FLAG-8xHis-GSGS-TEV_site_-sCT ZEO^R^ fms1-2∆* | This study |
| BSYBG10 *P_AOX1_-ScOST1-KpSCW10_pro_-FLAG-8xHis-GSGS-TEV_site_-sCT ZEO^R^ tCDC48* | This study |
| BSYBG10 *P_AOX1_-ScOST1-KpSCW10_pro_-FLAG-8xHis-GSGS-TEV_site_-αMSH ZEO^R^* | This study |
| BSYBG10 *P_AOX1_-ScOST1-KpSCW10_pro_-FLAG-8xHis-GSGS-TEV_site_- αMSH ZEO^R^ aqr1∆* | This study |
| BSYBG10 *P_AOX1_-ScOST1-KpSCW10_pro_-FLAG-8xHis-GSGS-TEV_site_- αMSH ZEO^R^ fms1-2∆* | This study |
| BSYBG10 *P_AOX1_-ScOST1-KpSCW10_pro_-FLAG-8xHis-GSGS-TEV_site_- αMSH ZEO^R^ tCDC48* | This study |

**Supplementary Table S4:** Components of the translocon expressed for increased 3×FLAG secretion

| **gene of interest** | **Identifier** |
| --- | --- |
| *SEC61* | [LT962476.2](https://www.ncbi.nlm.nih.gov/nucleotide/LT962476.2?report=genbank&log$=nuclalign&blast_rank=3&RID=WTJND72E014)- 377089..378525 |
| *SEC62* | [LT962478.2](https://www.ncbi.nlm.nih.gov/nucleotide/LT962478.2?report=genbank&log$=nuclalign&blast_rank=3&RID=WTJND72E014)- 293495..294346 |
| *SEC63* | [LT962479.2](https://www.ncbi.nlm.nih.gov/nucleotide/LT962479.2?report=genbank&log$=nuclalign&blast_rank=3&RID=WTJND72E014)- 994308..996302 |
| *KAR2* | [LT962477.2](https://www.ncbi.nlm.nih.gov/nucleotide/LT962477.2?report=genbank&log$=nuclalign&blast_rank=3&RID=WTJND72E014)- 2129225..2131261 |

**Supplementary Figure S1:** Comparison of commercial fossil- and carbon capture-derived methanol for the induction of secretory 3×FLAG production in *K. phaffii*. The Ost1_pre_-3×FLAG secretion strain was cultivated in shake flasks using BMD for biomass accumulation during a 60 h batch phase followed by a 24 h feed with methanol from two different sources. 3×FLAG in the culture supernatant was detected via immunoblot using Anti-FLAG antibody.

**Supplementary Figure S2:** Intact mass of the 3×FLAG peptides (average mass: 2861.9 Da) secreted by *K. phaffii* CBS7435 *his4* *tCDC48*. Shown are the monoisotopic peaks of the (A) peptide standard and the (B) secreted peptide after deconvolution. For the secreted 3×FLAG an additional, potentially oxidized species (+16 Da) was found.

**Supplementary Figure S3:** Intact mass of the α-MSH secreted by *K. phaffii* CBS7435 *his4* *fms1-2∆.* Shown are the monoisotopic peaks of the acetylated and amidated standard (average mass:1664.9 Da) and the oxidized secreted (average mass:1623.85 +32 Da) peptide after deconvolution.
